# Different *p53* genotypes regulating different phosphorylation sites and subcellular location of CDC25C associated with the formation of polyploid giant cancer cells

**DOI:** 10.1186/s13046-020-01588-w

**Published:** 2020-05-11

**Authors:** Kai Liu, Minying Zheng, Qi Zhao, Kexin Zhang, Zugui Li, Fangmei Fu, Hao Zhang, Jiaxing Du, Yuwei Li, Shiwu Zhang

**Affiliations:** 1 Department of Pathology, Tianjin Union Medical Center, Tianjin, 300121 P.R. China; 2 Graduate School, Tianjin Medical University, Tianjin, 300070 P.R. China; 3 Nankai University School of Medicine, Nankai University, Tianjin, 300071 P.R. China; 4 Graduate School, Tianjin University of Traditional Chinese Medicine, Tianjin, 300071 P.R. China; 5 Departments of Colorectal Surgery, Tianjin Union Medical Center, Tianjin, 300121 P.R. China

**Keywords:** Polyploid giant cancer cells, Cell cycle-related proteins, Phosphorylation, CDC25C, *p*53

## Abstract

**Background:**

Our previous studies have confirmed that cobalt chloride (CoCl_2_) can induce the formation of polyploid giant cancer cells (PGCCs), which is the key to the heterogeneity of solid tumors. PGCC formation is closely related to the abnormal expression of cell cycle-related proteins and cell fusion. In this study, we investigated the molecular mechanism of PGCCs formation by detecting the expression of cell cycle-related proteins in mutant and wild-type *p53* cancer cell lines.

**Methods:**

HEY, BT-549, SKOv3 and MDA-MB-231 cells were treated with CoCl_2_ and the cell cycle was detected by flow cytometry. The expression and subcellular localization of cell cycle-related proteins, kinases, and P53 were compared before and after CoCl_2_ treatment. Immunoprecipitation was used to analyze the interacting proteins of pCDC25C-Ser216 and pCDC25C-Ser198. The clinicopathologic significances of these cell cycle-related proteins and protein kinases expression were studied.

**Results:**

CoCl_2_ induced the formation of PGCCs and G2/M arrest. CDC25C, cyclin B1, and CDK1 expressions after CoCl_2_ treatment were lower than that in control cells. Cytoplasmic CDC25C was degraded by ubiquitin-dependent proteasome. The expression of P53 and phosphokinases including CHK1, CHK2, PLK1, and Aurora A increased after CoCl_2_ treatment. The expression of pCDC25C-Ser216 and pCDC25C-Ser198 depended upon the genotype of *p53*. The expressions of cell cycle-related proteins and kinases gradually increased with the development of ovarian cancer and breast cancer.

**Conclusion:**

CHK1, CHK2–pCDC25C-Ser216–cyclin B1–CDK1, and Aurora A–PLK1–pCDC25C-Ser198–cyclin B1–CDK1 signaling pathways may participate in the formation of PGCCs and different phosphorylation sites of CDC25C may be associated with the genotype of *p53*.

## Background

We previously reported that polyploid giant cancer cells (PGCCs) had the properties of cancer stem cells (CSCs) [1] and commonly appeared in many kinds of malignant tumor. PGCCs were once thought to be senescent cells due to their inability to undergo mitosis or cytokinesis and had no long-term survival or proliferative capacity [2]. Cobalt chloride (CoCl_2_), a chemical hypoxia simulator, was used to induce the formation of PGCCs in vitro. When recovered from CoCl_2_ treatment, PGCCs can generate daughter cells via asymmetric cell division. Daughter cells from PGCCs have strong invasion and metastatic abilities [1, 3]. In malignant solid tumor tissue, PGCCs often appear around necrotic areas and in the boundary of infiltration between normal and tumor tissues (invasive front), where tumor cells are under hypoxic microenvironment [4]. Our previous study has confirmed that PGCCs can be formed by cell fusion and that cell fusion-related proteins syncytin 1, CD9, and CD47 participate in PGCCs formation [5]. Furthermore, abnormal expression of cell cycle-related proteins and changes in subcellular localization also play an important role in the formation of PGCCs [1, 6].

PGCC formation is associated with abnormal expression and change in subcellular localization of CDC25C [6]. CDC25C plays an important role in cell division, proliferation, DNA damage and cell cycle arrest, serine/threonine kinase activity and mitotic cell G2/M transformation regulation [7–9]. Cell response to DNA damage is mainly coordinated by two different kinase signaling cascades, namely ataxia telangiectasia-mutated gene (ATM)–CHK2 and Rad3-related serine/threonine kinase (ATR)–CHK1 pathways, which are activated by DNA double and single strand breaks, respectively [10]. CDC25C is regulated by CHK1/CHK2 to induce G2/M phase arrest when DNA mismatch occurs [11]. It has been reported that tumor suppressor P53 cooperates with checkpoint proteins to regulate CDC25C and participate in G2/M arrest [12]. PLK1 and Aurora A, as members of the serine kinase family, participate in the regulation of CDC25C activation during mitosis [13]. The abnormal expression of CDC25C is regulated by CHK1, CHK2, PLK1, Aurora A, P53 and CDC25C phosphorylation at specific sites which determine its subcellular localization. Li et al. have reported that enhanced exogenous and functional P53 in *p*53-deficient cells decreased the total CDC25C and pCDC25CSer216 expression level, suggesting that DNA damage response (DDR) regulated CDC25C expression in a *p*53-dependent manner [11, 14]. In this study, the molecular mechanisms regulating CDC25C expression and subcellular location in HEY, BT-549, SKOv3 and MDA-MB-231cells before and after CoCl_2_ treatment were studied and the clinicopathological significance of CDC25C expression-related proteins were evaluated in human ovarian and breast cancer tissues.

## Methods

### Cancer cell lines and cultures

The human ovarian cancer cell line HEY (wild-type *p53*) and SKOv3 with lack of *p*53, human breast cancer cell line BT-549 (mutant-type *p*53) and MDA-MB-231 (mutant-type *p*53), were obtained from the American Type Culture Collection (Manassas, VA, USA) and the culture conditions were described previously [1].

### Induction, definition and counting of PGCCs

The induction, definition and counting of PGCCs were described previously [1]. HEY, SKOv3, BT-549 and MDA-MB-231 cells were incubated in T25 flasks with 1640 medium until they reached 90% confluence. They were treated with 450 μM CoCl_2_ (Sigma-Aldrich, St. Louis, MO, USA) for 24 and 48 h, respectively, based on their hypoxia-resistance abilities. Only scattered PGCCs survived after CoCl_2_ treatment and most regular-sized cancer cells were dead. Fifteen to twenty days later, PGCCs began to produce daughter cells via asymmetric division. After treatment with CoCl_2_ 3–4 times, the PGCCs occupied 20–30% total cells and 70–80% were the daughter cells derived from PGCCs. The cells treatment process was unified to ensure that the proportion of PGCCs and daughter cells were constant in this range. For the definition and counting of PGCCs, PGCCs were defined as tumor cells with a nucleus more than three times larger than that of diploid tumor cells, usually appearing as multinucleated and giant nucleated cells. Five microscopic fields were randomly counted at 400× magnification and the average PGCC percentage in slides was calculated after hematoxylin–eosin (H&E) staining. In this study, “control” represents the untreated control cells, “treatment” is used to indicate that HEY, BT-549, SKOv3 and MDA-MB-231cells with CoCl_2_ treatment.

### Transient siRNA transfection

CDC25C and P53 were knockdown by transient siRNA transfection. The siRNA oligonucleotides were synthesized by Gene-pharma (Shanghai, China), including three siRNA interference sequences, one positive control sequence (GAPDH), one negative control (NC) sequence, and one mock control (MC) with only transfection reagents. Three CDC25C transfection sequences including 1103, 1343, 1531and three P53 transfection sequences including 339, 886, 985 were used to inhibit the expression of CDC25C (Tables S1–S2). Based on the results of western blot, siRNA CDC25C-1531 and P53*–*339 were proved to have the strongest inhibitory efficiency and used in this study. Detailed information about transient siRNA transfection is provided in the Supplementary Materials and Methods.

### Total protein extraction and separation of cytoplasmic nuclear proteins

Total protein extraction and separation of cytoplasmic nuclear proteins were carried out as described previously [6]. The detailed information is provided in the Supplementary Materials and Methods.

### Western blot analysis

The protein concentrations from control and CDC25C-siRNA (CDC25Ci) transfection HEY and BT-549 cells before and after CoCl_2_ treatment were determined. β-actin was used as a protein-loading control, and all the western blot were duplicated at least three times. Detailed information about antibodies is provided in the Supplementary Materials and Methods and Tables S3.

### H&E staining

Control and CDC25C-siRNA (CDC25Ci) transfection HEY and BT-549 cells before and after CoCl_2_ treatment were cultured on slides for H&E staining. The detailed information about H&E staining is provided in the Supplementary Materials and Methods.

### Immunohistochemical (IHC) and immunocytochemical (ICC) staining

IHC and ICC staining was carried out as described previously [1]. Detailed information of IHC and ICC stain in is provided in the Supplementary Materials and Methods.

### Scoring and quantification of IHC

The protein expression was evaluated as previously described [5, 6]. Detailed information is provided in the Supplementary Materials and Methods.

### Cell migration and invasion assay

The abilities of cell migration and invasion assay were evaluated as previously described and detailed information about these assay is provided in the Supplementary Materials and Methods [6].

### Plate clone formation experiment

Plate clone formation experiment was evaluated as previously described [6]. Detailed information of plate clone formation experiment is provided in the Supplementary Materials and Methods.

### Tissue samples

Paraffin-embedded human ovarian tumor (*n* = 81) and breast cancer (*n* = 229) tissue samples were collected from the Department of Pathology in Tianjin Union Medical Center. All cases had complete clinicopathological data. The 81 human ovarian cancer tissues were divided into four groups: 20 primary ovarian cancer with lymph node metastasis (group I), 20 lymph node metastatic foci of group I (group II), 31 primary ovarian cancer without lymph node metastasis (group III), and 10 serious borderline serous cystadenoma (group IV). The 242 cases of human breast cancer were divided into two groups: 167 cases of invasive breast cancer without lymph node metastasis (group I) and 62 cases of breast cancer with lymph node metastasis (group II). The groups and clinical features of both ovarian tumor and breast cancer were listed in supplementary table 4. The Hospital Review Board of the Tianjin Union Medical Center approved this study and patient information confidentiality was maintained.

### MG-132 inhibitor treatment

The cells were cultured in a 24-well plate until they reached 80% confluence, then 10 μmol/L MG132 (Target Molecular, Boston, USA) was added in the wells for 6 h, followed by lysis buffer for western blot analysis.

### Flow cytometry analysis of cell cycle

Cell cycle status was detected by flow cytometry and the detailed information is provided in the Supplementary Materials and Methods.

### Vitro kinase activity assay

The kinase activity assay was performed using the EnzyChrom Kinase Assay Kit (BioAssay Systems, USA) to test the differences in protein kinase activity before and after CoCl_2_ treatment in vitro. Detailed information about kinase activity assay is provided in the Supplementary Materials and Methods.

### Co-immunoprecipitation (co-IP)

Co-IP was performed to determine the direct or indirect interaction with pCDC25CSer216 and pCDC25CSer198 in HEY and BT-549 cells after CoCl_2_ treatment. The detailed information about Co-IP is provided in the Supplementary Materials and Methods.

### Statistical analysis

SPSS 22 (SPSS Inc., Chicago, USA) was used for all statistical analysis in this study. All histogram data are expressed as mean ± SD, and all tabular data are presented as mean ± SEM. Kruskal-Wallis test was performed to compare the differences in cell cycle-related protein expressions in ovarian cancer tissues. The Mann-Whitney test was used to analyze the differences in cell cycle-related protein expressions in breast cancer tissues. Other comparisons were performed using a two-tailed Student’s *t*-test and Pearson chi-square (χ^2^) test. A two-tailed *P* value less than 0.05 was defined as statistically significant.

## Results

### Formation of PGCCs following CoCl_2_ treatment

**Fig. 1:**
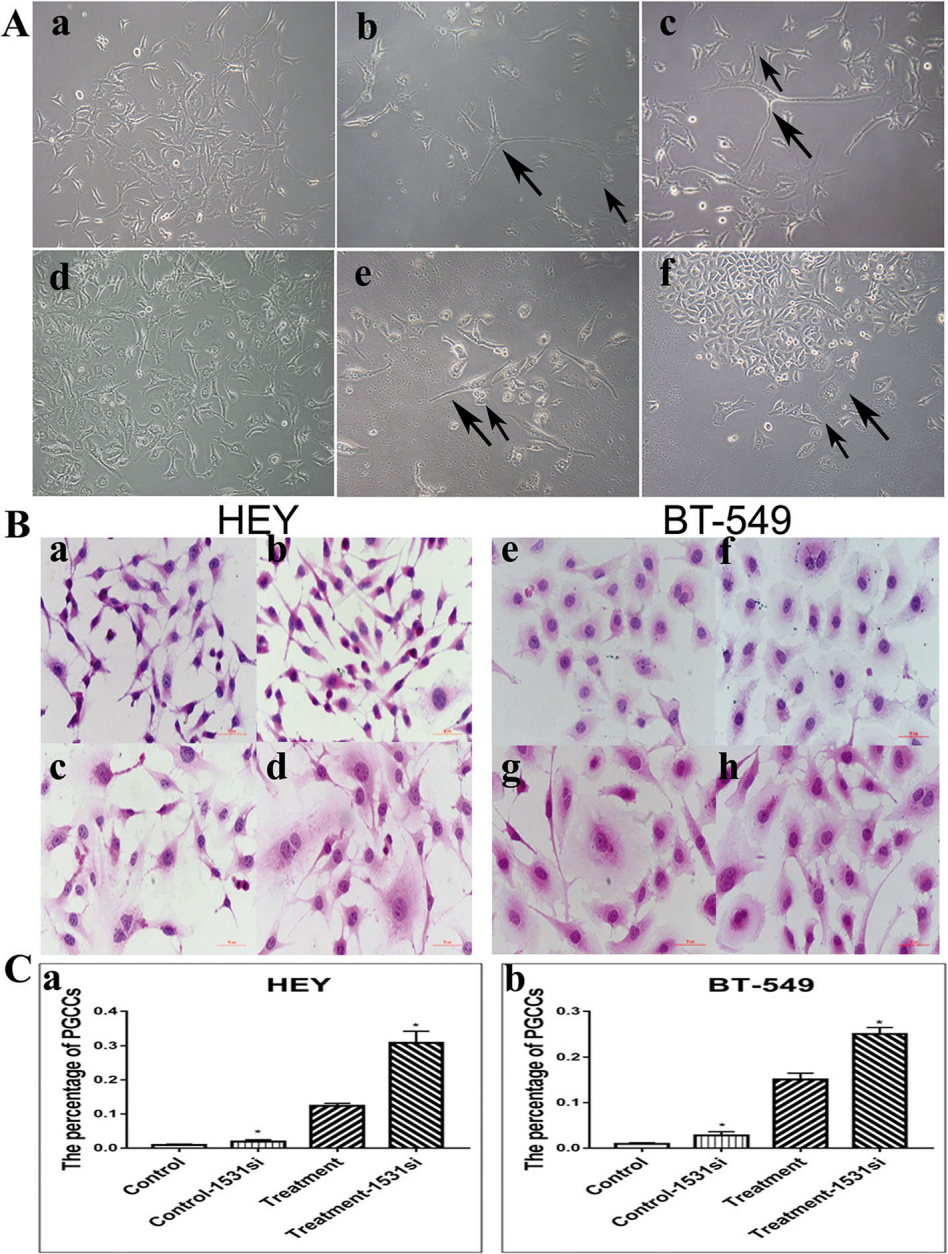
PGCCs with budding daughter cells in HEY and BT-549 cells. **a** HEY and BT-549 control cells and PGCCs. (a) HEY control cells, (b) HEY PGCCs induced by 450 μM CoCl_2_ treatment for 48 h, (c) PGCCs and their daughter cells; the large black arrow indicates PGCCs and the small black arrow heads the daughter cells, (d) BT-549 control cells, (e) BT-549 PGCCs induced by 450 μM CoCl_2_ treatment for 24 h, and (f) PGCCs and their daughter cells; the large black arrow indicates PGCCs and the small black arrow heads the daughter cells. **b** H&E staining of the HEY and BT-549 cells before and after CDC25i. (a) HEY control cells, (b) Control cells after CDC25C knockdown, (c) HEY PGCCs with daughter cells, (d) HEY PGCCs and daughter cells after CDC25C knockdown, (e) BT-549 control cells, (f) Control cells after CDC25C knockdown, (g) H&E staining of the BT-549 PGCCs with daughter cells, and (h) BT-549 PGCCs with daughter cells after CDC25C knockdown **c** (a) The percentage of HEY PGCCs in control, control cells after CDC25i, PGCCs with daughter cells, and PGCCs with daughter cells after CDC25Ci. (b) The percentage of BT-549 PGCCs in control, control cells after CDC25i, PGCCs with daughter cells, and PGCCs with daughter cells after CDC25Ci. All magnifications are at 100×. Treatment: Cells treated with CoCl_2_. 1531si: siRNA CDC25C-1531

When high concentration (450 μM) of CoCl_2_ was added to HEY(Fig. 1A a) for 48 h and BT-549 (Fig. 1A d) for 24 h, most regular-sized diploid cells were killed and only few PGCCs survived the CoCl_2_ treatment (Fig. 1A b, e). The surviving PGCCs could generate daughter cells via asymmetric division (Fig. 1A c, f). Furthermore, to investigate whether CDC25C knockdown affects PGCCs formation, H&E staining was used to count the number of PGCCs in control cells (Fig. 1B a, e) and PGCCs with their daughter cells (Fig. 1B c, g), as well as their CDC25C-siRNA (CDC25Ci) groups. According to the statistical results showed in Table S5, the number of PGCCs in HEY and BT-549 after CoCl_2_ treatment was higher than that in control cells. There also were more PGCCs in CDC25Ci group (Fig. 1B b, d, f, h) than in the negative control group (Fig. 1B a, c, e, g). The differences among these groups were statistically significant (Fig. 1C a, b). Thus, CoCl_2_ treatment and CDC25C knockdown can induce the formation of PGCCs.

### CDC25C participates in PGCCs formation by regulating cyclin B1-CDK1 complex

**Fig. 2:**
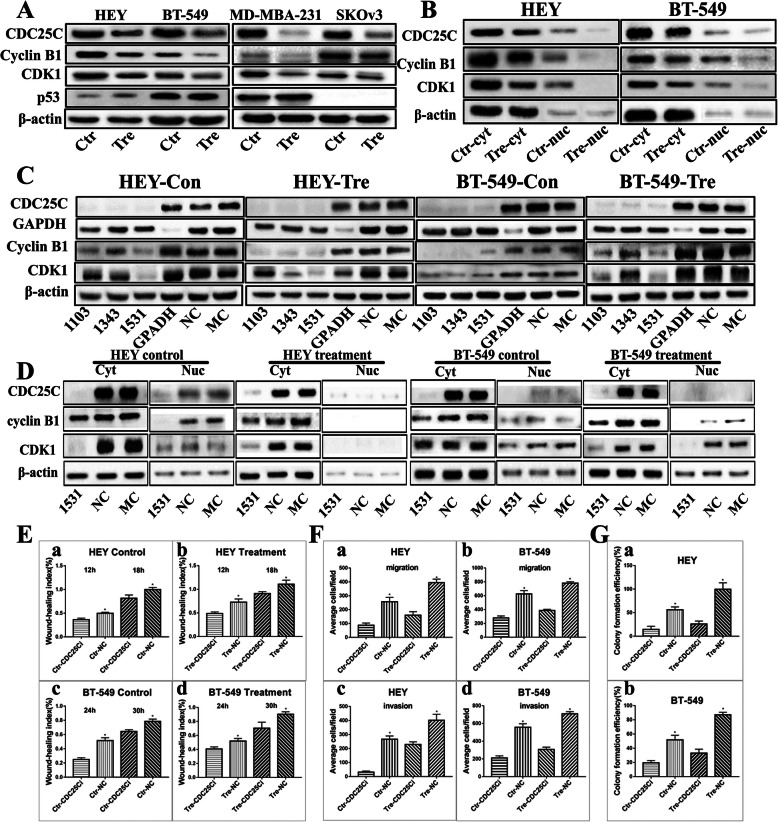
CDC25C, cyclin B1, and CDK1 expression in HEY, SKOv3, BT-549 and MDA-MB-231 cells before and after CoCl_2_ treatment. **a** Western blot showed the total protein expression of CDC25C, cyclin B1, and CDK1 in HEY, SKOv3, BT-549 and MDA-MB-231 control and PGCCs with daughter cells. **b** Western blot results show CDC25C, cyclinB1, and CDK1 cytoplasmic and nuclear expression in HEY and BT-549 control and PGCCs with daughter cells. **c** Total protein expression of CDC25C, cyclin B1, and CDK1 in HEY control and PGCCs with daughter cells with siRNA CDC25C-1103, 1343, 1531, siRNA control, and negative control transfection. **d** CDC25C, cyclin B1 and CDK1 cytoplasmic and nuclear expression in HEY and BT-549 control and PGCCs with daughter cells with siRNA CDC25C-1531, siRNA control, and negative control transfection. **e** Histogram was used to quantify wound-healing index in HEY and BT-549 cells by measuring no less than three different healing areas. (a) HEY control cells treated with CDC25C-siRNA and siRNA control. (b) HEY PGCCs and daughter cells treated with CDC25C-siRNA and siRNA control. (c) BT-549 control cells treated with CDC25C-siRNA and siRNA control. (d) BT-549 PGCCs and daughter cells treated with CDC25C-siRNA and siRNA control. **f** Column diagram of the average cell number for migration and invasion assay in HEY and BT-549 control, PGCCs, and daughter cells after the CDC25C-siRNA and siRNA control transfection. Average cell number for migration among (a) HEY and (b) BT-549 cells after the CDC25C-siRNA and siRNA control transfection. Average cell number for invasion among (c) HEY and (d) BT-549 cells after the CDC25C-siRNA and siRNA control transfection. **g** Column diagram shows the colony formation efficiency in HEY and BT-549 control and PGCCs with daughter cells after CDC25C-siRNA and siRNA control transfection. Colony formation efficiency of (a) HEY and (b) BT-549 with different treatment. Treatment: Cells treated with CoCl_2_. CDC25i: CDC25C-1531 siRNA

In order to explore whether CDC25C is related with PGCCs formation by regulating cyclin B1–CDK1 complex, CDC25C was knocked down by transient transfection. Western blot were used to verify CDC25C, cyclin B1, and cyclin-dependent protein kinases 1 (CDK1) expression levels and subcellular localization. The average number of PGCCs in 5 high-power-fields (400×) occupied 28% of the total cell and 72% was the daughter cells based on the H&E staining. Western blot results showed that the total protein level of CDC25C, cyclin B1 CDK1 and decreased after CoCl_2_ treatment in HEY, BT-549, SKOv3 and MDA-MB-231 cells compared with those in control cells (Fig. 2A). Results of quantitative analysis showed remarkable differences of CDC25C, cyclinB1, CDK1 expression before and after CoCl_2_ treatment (Fig.S1 a-c). Subsequently, cytoplasmic and nuclear protein separation was performed to detect CDC25C, cyclin B1, and CDK1 subcellular localizations (Fig. 2B and S1 d-f). Both the cytoplasm and nucleus of HEY and BT-549 cells can express CDC25C, cyclin B1, and CDK1 and the expression of these proteins was higher in the cytoplasm than that in the nucleus of the control cells. After CoCl_2_ treatment, CDC25C, cyclin B1, and CDK1 was detected mainly in the cytoplasm of HEY and BT-549 cells. After CDC25C knockdown, the expression of cyclin B1 and CDK1 in HEY and BT-549 control cells, and cells after CoCl_2_ treatment decreased compared with those in siRNA control groups, NC and MC (Fig. 2C). These results indicated that CDC25C expression was positively correlated with that of cyclin B1 and CDK1, and cyclin B1 and CDK1 cytoplasmic expression levels significantly decreased after CDC25C knockdown (Fig. 2D).

### Knockdown of CDC25C decreased cancer cell migration, invasion, and proliferation abilities

Cells after CDC25Ci (siRNA CDC25C-1531) treatment for 24–48 h were used to measure the migration ability of cells in control, PGCCs with their daughter cells, control-CDC25Ci, and PGCCs with daughter cells-CDC25Ci by wound-healing assay. The average number of PGCCs in 5 high-power-fields (400×) occupied 25% of the total cell and 75% was the daughter cells based on the H&E staining. Fig. S2A shows the results of wound-healing assay for HEY at 0, 12, and 18 h and BT-549 at 0, 24, and 30 h. The migration ability of cells in CDC25Ci group was lower than that in the corresponding negative control group. The migration ability of PGCCs with their daughter cells was stronger than that in control group (Fig. 2E a-d). Transwell migration assay showed that the number of cells migrated in CDC25Ci group was lesser than that of the negative control group for HEY at 12 h and BT-549 at 24 h (Fig. 2F a, b and Fig. S2B a-d and S2C a-d). Cell invasion assay was performed using matrigel-coated transwell inserts. Fig. S2B e-h and S2C e-h reveals that the invasion ability in CDC25Ci was lower than that in the negative control group. Our results proved that CDC25Ci inhibited HEY and BT-549 cell invasion abilities (Fig. 2F c, d). Plate cloning assay was used to detect the cell proliferative ability. The numbers of clones formed in 50, 100, and 200 HEY and BT-549 cells-CDC25Ci were less than those of 50, 100, and 200 negative control cells (Fig. 2G and Fig. S2D a–d). Compared with cells without CDC25C knockdown, CDC25C knockdown could decrease the abilities of migration, invasion, and proliferation in control, PGCCs with their daughter cells, and the differences were statistically significant (Tables S6–S10).

### Expression of Aurora A-PLK1-pCDC25CSer198 and CHK1/CHK2- pCDC25CSer216 in HEY and BT-549

**Fig. 3:**
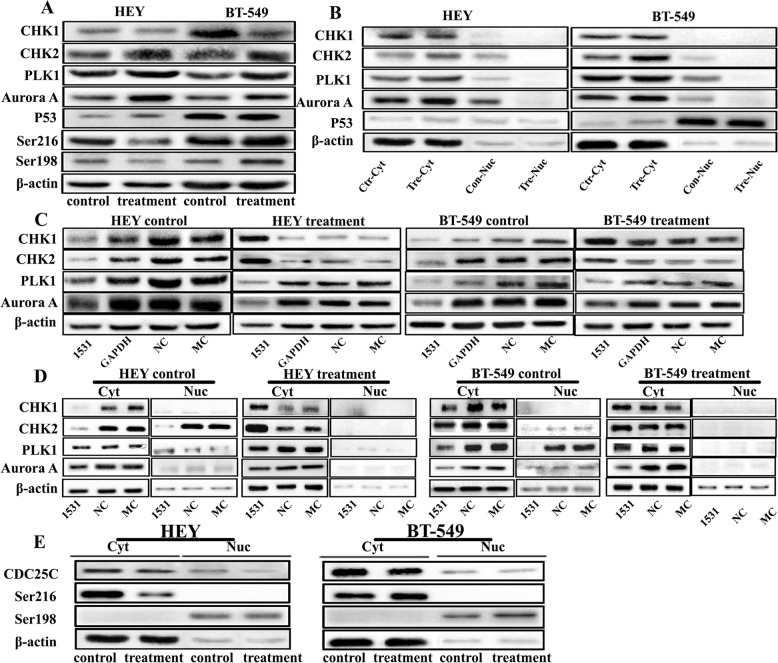
The expression of CHK1, CHK2, PLK1, Aurora A, P53, pCDC25CSer216, and pCDC25CSer198 in HEY and BT-549 control cells and PGCCs with budding daughter cells with and without CDC25C knockdown. **a** Western blot showed the total protein expression of CHK1, CHK2, PLK1, Aurora A, P53, pCDC25CSer216, and pCDC25CSer198 in HEY and BT-549 control and PGCCs with daughter cells. **b** The levels of total protein expression of CHK1, CHK2, PLK1, and Aurora A in HEY and BT-549 control and PGCCs with daughter cells, which were transfected with CDC25Ci, siRNA control, and negative control. **c** Cytoplasmic and nuclear protein expression of CHK1, CHK2, PLK1, Aurora A, P53 in HEY and BT-549 control and PGCCs with daughter cells. **d** Cytoplasmic and nuclear expression of CHK1, CHK2, PLK1, and Aurora A in HEY and BT-549 control and PGCCs with daughter cells, which were transfected with CDC25Ci, siRNA control, and negative control. **e** The cytoplasm and nuclear protein expression of pCDC25CSer216 and pCDC25CSer198 in HEY and BT-549 control and PGCCs with daughter cells. Treatment: Cells treated with CoCl_2_. 1531si: siRNA CDC25C-1531

CHK2, PLK1, Aurora A, and P53 total protein levels increased in HEY and BT-549 PGCCs with their daughter cells(Fig. 3A and Fig. S3A a-e). However, CHK1 showed a slight decrease in PGCCs with their daughter cells compared to the control cells. The expression of pCDC25CSer216 and pCDC25CSer198 in HEY PGCCs with their daughter cells decreased compared with them in the control. However, these two phosphorylation proteins increased in BT-549 PGCCs with their daughter cells (Fig. 3A and Fig. S3A f-g). Based on the results of western blot, siRNA CDC25C-1531 was proved to have the strongest inhibitory efficiency and was chosen in this part. In CDC25Ci group of HEY and BT-549 before and after CoCl_2_ treatment, PLK1 and Aurora A expression decreased compared to that in the cells without CDC25C knockdown. However, the expression levels of CHK1 and CHK2 in the control group were lower than them in the siRNA control. Also, CDC25C expression inhibition increased CHK1 and CHK2 expression in PGCCs with their daughter cells (Fig. 3C), which suggested that the expression levels of CHK1 and CHK2 as checkpoint kinases were lower in normal mitotic cells than in PGCCs, which produced the daughter cells via asymmetric cell division.

The results of cytoplasmic and nuclear proteins separation experiment showed that CHK1 was localized only in the cytoplasm of HEY and BT-549 after CoCl_2_ treatment, and there was no difference in the cytoplasmic expression levels before and after the treatment. CHK2, PLK1, and Aurora A were expressed both in the cytoplasm and nucleus in control cells and the cytoplasmic expression was higher than the nuclear expression. In PGCCs with their daughter cells, CHK2, PLK1, and Aurora A were only located in the cytoplasm and the expression levels were higher compared with those in the control cells (Fig. 3B). Before and after CoCl_2_ treatment, P53 was almost undetectable in HEY, but was detected only in the nucleus of BT-549 cells (Fig. 3B and Fig. S3B a). In CDC25Ci cells, cytoplasmic CHK1 and CHK2 were highly expressed in CoCl_2_-treated cells and poorly expressed in control cells. PLK1 and Aurora A cytoplasmic expression was decreased both in HEY and BT-549 cells after CDC25C knockdown before and after CoCl_2_ treatment. Furthermore, PLK1 and Aurora A expression in the nucleus of control cells decreased after CDC25Ci. The nucleus of PGCCs with daughter cells after CDC25Ci treatment did not express PLK1 and Aurora A (Fig. 3D).

Cytoplasm and nuclear protein separation were also used to detect the expression and subcellular location of pCDC25CSer216 and pCDC25CSer198. The expression of pCDC25CSer216 was located in the cytoplasm and pCDC25CSer198 in the nucleus of HEY and BT-549 before and after CoCl_2_ treatment (Fig. 3E). The expression of pCDC25CSer216 decreased in HEY PGCCs with their daughter cells and increased in BT549 PGCCs with their daughter cells, compared with that in the control cells (Fig. S3B b-c). Moreover, pCDC25CSer198 nuclear expression of PGCCs with their daughter cells decreased in HEY and increased in BT-549 compared to that in control cells.

**Fig. 4:**
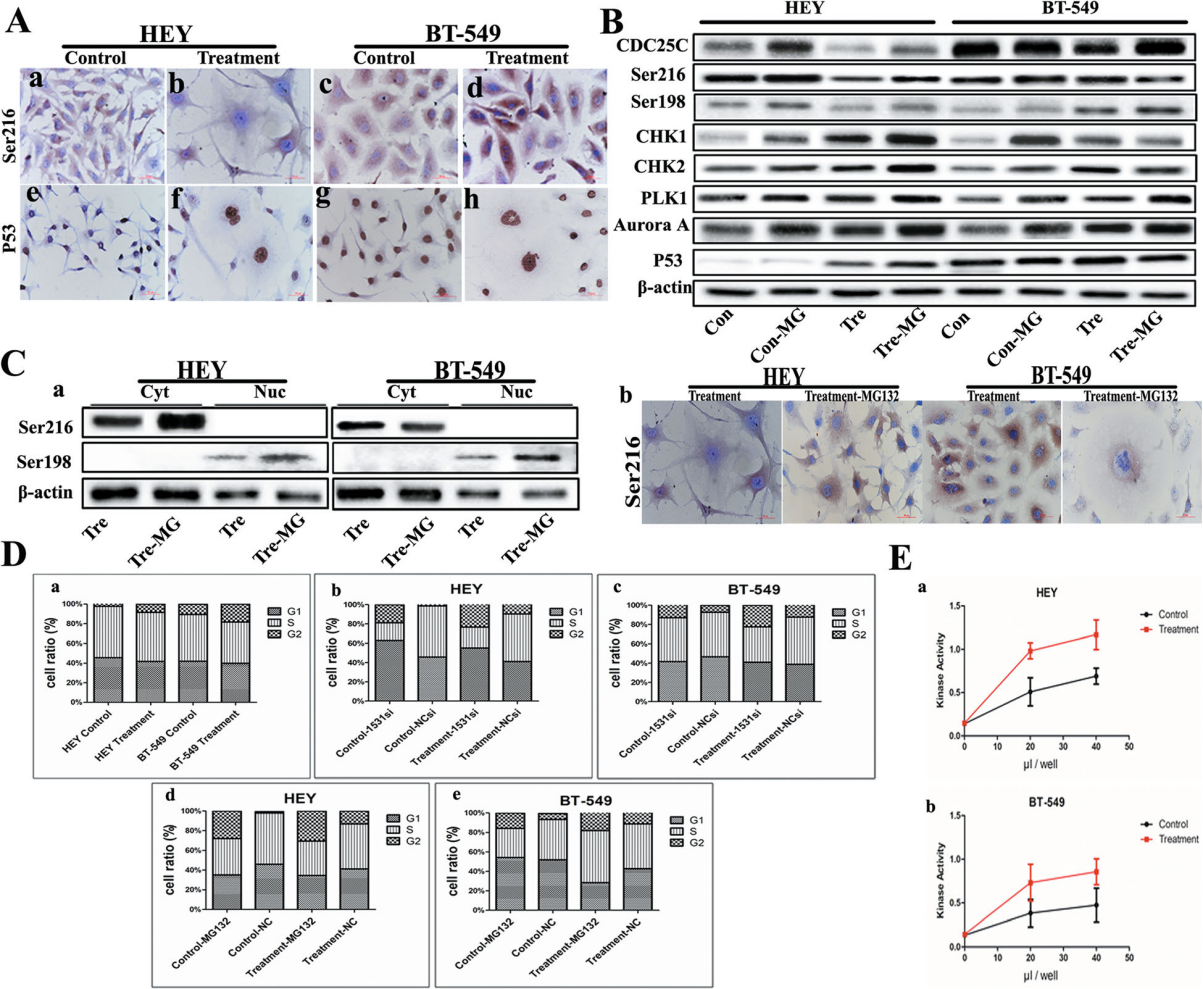
**a** ICC staining of pCDC25CSer216 and P53 in HEY and BT-549 before and after CoCl_2_ treatment (200×). pCDC25CSer216 in (a) HEY control cells, (b) HEY PGCCs with daughter cells, (c) BT-549 control, and (d) BT-549 PGCCs with daughter cells. P53 in (e) HEY control, (f) HEY PGCCs with daughter cells, (g) BT-549 control, and (h) BT-549 PGCCs and daughter cells. **b** CDC25C, pCDC25CSer216, pCDC25CSer198, CHK1, CHK2, PLK1, Aurora A, P53 expression in HEY and BT-549 control cells and PGCCs before and after MG-132 treatment. **c** (a) Cytoplasmic and nuclear expression of pCDC25CSer216 and pCDC25CSer198 in HEY and BT-549 control cells and PGCCs after MG-132 treatment. (b) ICC staining showing the subcellular localization of pCDC25CSer216 in HEY and BT-549 PGCCs with budding cells after MG-132 treatment (400×). **d** Columnar percentage plot showing the ratio of cells at G1, S, and G2 stages of cell cycle in HEY and BT-549 cells. (a) The ratio of cells at G1, S, and G2 stages in HEY control and PGCCs with daughter cells before and after CoCl_2_ treatment. (b) The ratio of cells at G1, S, and G2 stages in HEY control and PGCCs with daughter cells before and after CDC25C knockdown. (c) The ratio of cells at G1, S, and G2 stages in BT-549 control and PGCCs with daughter cells before and after CDC25C knockdown. (d) The ratio of cells at G1, S, and G2 stages in HEY control and PGCCs with daughter cells before and after MG132 treatment. (e) The ratio of cells at G1, S, and G2 stages in BT-549 control and PGCCs with daughter cells before and after MG132 treatment. **e** The results of kinase activity assay in vitro. (a) HEY control cells and PGCCs with budding. (b) BT-549 control cells and PGCCs with budding. Treatment: Cells treated with CoCl_2_. MG: Cells were treated with MG-132. 1531si: siRNA CDC25C-1531

As shown in Fig. S4, CHK1 was detected only in cytoplasm in HEY and BT-549 cells before and after CoCl_2_ treatment (Fig. S4A a, c and S4B a, c). The cytoplasmic expressions of CHK2, PLK1, and Aurora A were higher than those in nucleus, which further increased after CoCl_2_ treatment (Fig. S4A e, g, i, k, m, o and S4B e, g, i, k, m, o). In HEY and BT-549 cells treated with CDC25Ci, results of ICC staining for CHK1, CHK2, PLK1, and Aurora A were consistent with western blot results. CHK1 cytoplasmic expression decreased in HEY and BT-549 cells after CDC25C knockdown compared to those in the cells without CDC25Ci (Fig. S4A b, d and S4B b, d). CHK2 (Fig. S4A f, h and S4B f, h), PLK1 (Fig. S4A j, l and S4B j, l), and Aurora A (Fig. S4A n, p and S4B n, p) were expressed both in cytoplasm and nucleus and also decreased in cells with CDC25Ci. pCDC25CSer216 expression was only detected in the cytoplasm and decreased in HEY PGCCs with daughter cells compared with the control cells (Fig. 4A a, b). However, pCDC25CSer216 was highly expressed in BT-549 PGCCs with daughter cells compared with that in control cells (Fig. 4A c, d). There was no expression of P53 in HEY control cells (Fig. 4A e). It is interesting that P53 expression could be detected in HEY after CoCl_2_ treatment (Fig. 4A f), which showed that CoCl_2_ might induce *p53* genotype transformation. P53 expression appeared in BT-549 both before and after CoCl_2_ treatment (Fig. 4A g, h).

### Cell cycle-related protein expression after MG132 treatment

HEY and BT-549 cells were treated with ubiquitin-mediated proteasome inhibitor MG132, which can inhibit ubiquitin-dependent proteasome and increase CDC25C expression. Western blot results showed that CDC25C expression increased in HEY and BT-549 cells after MG132 treatment, suggesting that proteasome degradation pathway may be involved in CDC25C expression. In this part of the experiment, the average number of PGCCs in 5 high-power-fields (400×) occupied 27% of the total cell and 73% was the daughter cells based on the H&E staining. In addition, pCDC25CSer216, pCDC25CSer198, CHK1, CHK2, PLK1, Aurora A, and P53 expressions increased in HEY control cells and PGCCs after MG132 treatment. In BT549 PGCCs with daughter cells after MG132 treatment, pCDC25CSer216, CHK1, and CHK2 expressions decreased and pCDC25CSer198, PLK1, Aurora A, and P53 expressions increased compared with those in the control cells (Fig. 4B). Western blot results for cytoplasmic and nuclear protein separation indicated that pCDC25CSer216 cytoplasmic expression increased in HEY PGCCs after MG132 treatment and decreased in BT-549 PGCCs (Fig. 4C a). ICC results revealed a significantly increased and decreased cytoplasmic expression of pCDC25CSer216 in HEY and BT-549 PGCCs, respectively, after MG132 treatment (Fig. 4C b).

### Cell cycle analysis in cells after CoCl_2_ and MG132 treatment

Results of cell cycle analysis revealed that there were more cells in G2/M phase in CoCl_2_-treated HEY and BT-549 cells (8.42 and 18.30% for HEY and BT-549 PGCCs, respectively) than in untreated control cells (2.18 and 10.63% for HEY and BT-549 control cells, respectively) (Fig. 4D a and Fig. S5A). Analysis for DNA content of HEY and BT-549 cells before and after CoCl_2_ treatment showed that cells with 4 N DNA content were more abundant in cells with CDC25Ci than in siRNA control cells, suggesting that CDC25Ci blocked cell cycle progression in G2/M phase (Fig. 4D b, c and Fig. S5B). Similar results could be observed in MG132-treated cells. The number of cells in G2/M phase increased in MG132-treated cells than in untreated cells, which showed that MG132 might play an important role in G2/M phase transition (Fig. 4D d, e and Fig. S5C). Kinase activity assay showed that the kinase activity of CoCl_2_-treated cells was higher than that in control cells, which showed that protein kinases might be involved in the formation of PGCCs (Fig. 4E a and b).

### Co-immunoprecipitation (co-IP) of pCDC25CSer216 and pCDC25CSer198

**Fig. 5:**
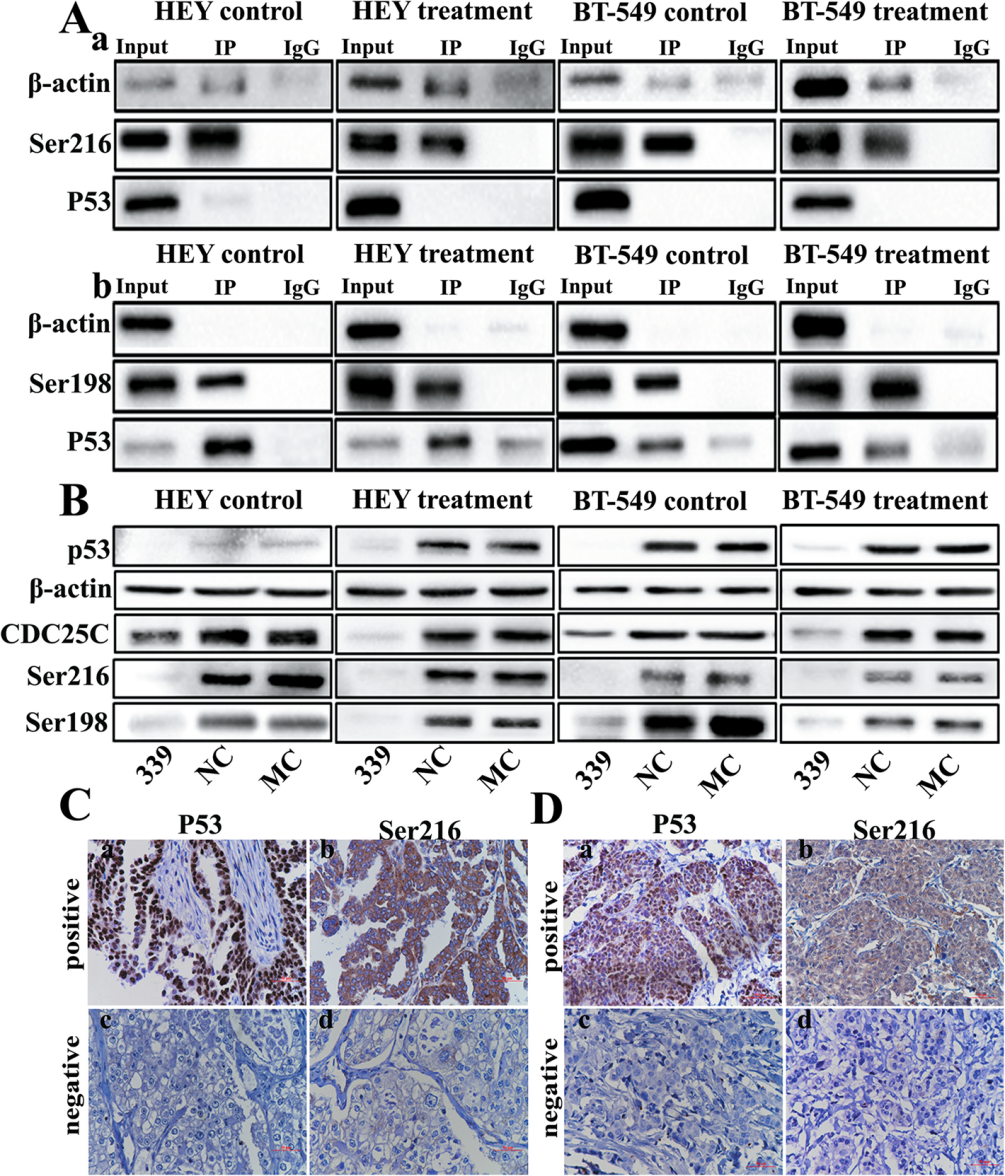
**a** Co-IP shows the interaction between pCDC25CSer216, pCDC25CSer198, and P53 in HEY and BT-549 cells. Western blot showed the interaction between (a) pCDC25CSer216 and P53 and (b) pCDC25CSer198 and P53. **b** The levels of total protein expression of CDC25C, pCDC25CSer216, and pCDC25CSer198 in HEY and BT-549 control and PGCCs with daughter cells transfected with *p53*i, siRNA control, and negative control. **c** Expression of P53 and pCDC25CSer216 in human breast cancer (IHC, × 100). Breast cancer tissue with (a) P53 and (b) pCDC25CSer216 positive expressions. Breast cancer tissue with (c) P53 and (d) pCDC25CSer216 negative expressions. **d** Expression of P53 and pCDC25CSer216 in human ovarian tumor (IHC, 100×). Ovarian cancer tissue with (a) P53 and (b) pCDC25CSer216 positive expressions. Ovarian carcinoma with (c) P53 and (d) pCDC25CSer216 negative expressions. Treatment: Cells treated with CoCl_2_. 339: siRNA *p*53–339

The expression and subcellular localization of pCDC25CSer216 and pCDC25CSer198 were different between HEY and BT-549 cells, which may be related to the different *p53* genotype in HEY and BT-549. Results of immunoprecipitation assay indicated that P53 interact with pCDC25CSer216 and pCDC25CSer198, regulating CDC25C expression through phosphorylating the Ser216 and Ser198 sites, respectively (Fig. 5A a, b).

### The expression of pCDC25CSer216 and pCDC25CSer198 in cells with *p*53 knockdown

The expression of CDC25C, pCDC25CSer216, and pCDC25CSer198 were detected in HEY and BT-549 cells with P53 knockdown. CDC25C, pCDC25CSer216, and pCDC25CSer198 expressions of HEY and BT-549 before and after CoCl_2_ treatment decreased after P53 knockdown. The decrease of pCDC25CSer216 and pCDC25CSer198 expression was more obvious than that of the total CDC25C expression, indicating that P53 can directly regulate pCDC25CSer216 and pCDC25CSer198 expressions (Fig. 5B).

Results of IHC staining analysis showed that P53 and pCDC25CSer216 expressions in the primary ovarian and breast cancer tumors were highly associated in multiple cases, as shown in Fig. 5C and D. According to statistical results (Table 1), P53 and CDC25CSer-216 were positively and negatively expressed in 25 and 13 cases of 51 ovarian carcinomas, respectively. Both positive and negative expression between P53 and CDC25CSer-216 in ovarian tumor had statistical significance (χ^2^ = 12.31, *P =* 0.000). In breast cancer, P53 and CDC25CSer-216 were positively and negatively expressed in 97 and 35 cases of 167 samples, respectively. Both positive and negative expression between P53 and CDC25CSer-216 in breast cancer had statistical significance (χ2 = 47.68, *P =* 0.000).

**Table 1:** Association of P53 and CDC25C-Ser216 expression in 51 cases of ovarian tumor and 167 cases of breast cancer

Ovarian tumor tissues		P53			χ2	*P*	Breast cancer		P53			χ2	*P*
		+	-	Total					+	-	Total		
CDC25C-Ser216	+	25	10	35	12.31	0.000	CDC25C-Ser216	+	97	9	106	47.68	0.000
	-	3	13	16				-	26	35	61		
	Total	28	23	51				Total	123	44	167		

### Expression of cell cycle protein in human breast tumor tissues and ovarian tumor tissues

**Fig. 6:**
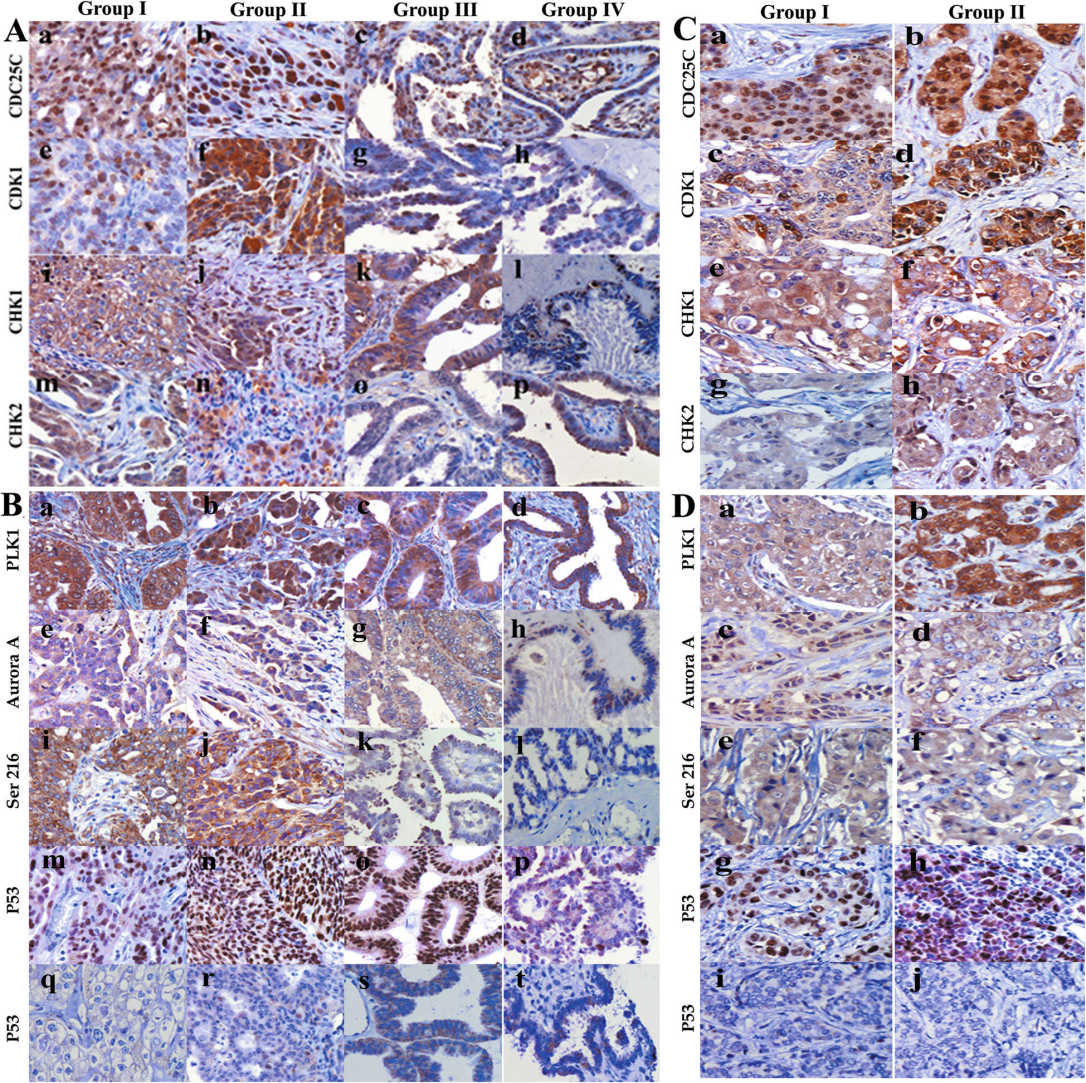
**a** Expression of cell cycle-related proteins in primary ovarian cancer with lymph node metastasis (group I), corresponding lymph node metastases (group II), primary ovarian cancer tissue without lymph node metastasis (group III), and serious borderline cystadenoma (group IV) (IHC, 200×). (a–d) CDC25C, (e–h) CDK1, (i–l) CHK1, and (m–p) CHK2 expressions in different groups. **b** Expression of protein kinase and P53 in primary ovarian cancer with lymph node metastasis (group I), corresponding lymph node metastases (group II), primary ovarian cancer tissue without lymph node metastasis (group III), and serious borderline cystadenoma (group IV) (IHC, 200×). (a–d) PLK1, (e–h) Aurora A, (i–l) pCDC25C-Ser216, and (m–p) positive and (q–t) negative P53 expressions in different groups. **c** Expression of cell cycle-related proteins in primary breast cancer (group I) and lymph node metastatic breast cancer (group II) (IHC, 200×). (a–b) CDC25C, (c–d) CDK1, (e–f) CHK1, and (g–h) CHK2 expressions in different groups. **d** Expression of protein kinase and P53 in primary breast cancer (group I) and lymph node metastatic breast cancer (group II) (IHC, 200×). (a–b) PLK1, (c–d) Aurora A, (e–f) pCDC25C-ser216, and (g–h) positive and (i–j) negative P53 expressions in different groups

IHC staining of cell cycle-related proteins expression were performed in 81 ovarian cancer and 238 breast cancer tissues (Fig. 6 A, B, C, D). The expression level of CDC25C increased in ovarian tumor and breast cancer cells, which are mainly composed of diploid tumor cells. Both the cytoplasm and nucleus of ovarian and breast cancer cells can express CDK1, CHK2, PLK1, and Aurora A. CHK1 and pCDC25CSer216 were located only in the cytoplasm. The differences of CDC25C (*P* = 0.000), pCDC25CSer216 (*P* = 0.001), CDK1 (*P* = 0.000), CHK1 (*P* = 0.000), CHK2 (*P* = 0.000), PLK1 (*P* = 0.000) and Aurora A (*P* = 0.000) expression had statistical significances among these ovarian tumor groups. For breast cancer, the differences of CDC25C (*P* = 0.001), CDK1 (*P* = 0.000), CHK1 (*P* = 0.001), CHK2 (*P* = 0.000), PLK1 (*P* = 0.000) and Aurora A (*P* = 0.000) expression between primary cancer with and without metastasis also had statistical differences (Tables S11–S16).

We also compared the difference of cell cycle-related proteins expression in different ovarian tumor groups. The results showed that CDC25C (*P* = 0.001), pCDC25CSer216 (*P* = 0.001), CDK1 (*P* = 0.000), CHK1 (*P* = 0.014), CHK2 (*P* = 0.000), PLK1 (*P* = 0.000), and Aurora A (*P* = 0.003) in lymph node metastatic carcinoma was significantly higher than that in primary carcinoma with metastatic lymph nodes and the difference was statistically significant. CDC25C (*P* = 0.015), pCDC25CSer216 (*P* = 0.004), CDK1 (*P* = 0.005), CHK1 (*P* = 0.030) and CHK2 (*P* = 0.000) expressions increased in the primary carcinoma with metastatic lymph nodes compared to those in primary carcinoma without metastasis, except for PLK1 (*P* = 0.199) and Aurora A (*P* = 0.194). In addition, the expression of CDC25C (*P* = 0.000), pCDC25CSer216 (*P* = 0.000), CDK1 (*P* = 0.000), CHK1 (*P* = 0.000), CHK2 (*P* = 0.000), PLK1 (*P* = 0.000) and Aurora A (*P* = 0.008) between the primary carcinoma without metastasis and serious borderline cystadenoma were also statistically different.

## Discussion

PGCCs are the key contributors to cancer heterogeneity, which can generate daughter cells with strong invasive ability via asymmetric division. PGCCs are the commonest histopathological characteristics of human malignant tumors. The number of PGCCs in high malignant tumor, tumor with lymph node metastasis, and recurrent tumor is more than that in low degree malignant tumor, tumor without lymph node metastasis, and primary tumor. Tumor tissue with more PGCCs has strong resistance to radiotherapy and chemotherapy [1]. Our previous studies showed that CoCl_2_ could induce the formation of PGCCs via endoreduplication and cell fusion. Variations of the mitotic cell cycle can occur under stresses, and endocycle (or endoreduplication) is a kind of variations which the normal mitotic cell cycle involves multiple rounds of DNA replication without an intervening mitosis step. In addition, cell fusion can contribute to the formation of PGCCs and play an important role in cancer progression [5]. In this study, flow cytometry analysis indicated that there were more G2/M phase cells in PGCCs than in control cells. G2/M arrest may be associated with the abnormal expression and subcellular location change of cell cycle-related proteins. CDC25C enters the nucleus in mitotic prophase and then shuttles back and forth between the cytoplasm and nucleus. The timely transfer of CDC25C from cytoplasm to nucleus is a critical step to enter mitosis [15]. During the interphase of cell division, phosphorylated CDC25C-Ser216 specifically binds to 14–3-3 protein, which confines CDC25C in the cytoplasm and inhibits its activity [16, 17]. Before mitosis, CDC25C dissociates from the 14–3-3 protein and activates cyclin B1–CDK1 complex, and enters the nucleus. Activated cyclin B1–CDK1 complex in turn activates CDC25C. Our previous results showed that CDC25C plays an important role in PGCCs formation [6]. In this study, we showed that different *p*53 genotypes in different cell lines may be associated with the formation of PGCCs by regulating the expression and subcellular location of CDC25C. CDC25C knockdown can induce the formation of PGCCs. Daughter cells derived from PGCCs had strong abilities of migration, invasion and proliferation which can be inhibited by CDC25C knockdown. IHC staining confirmed that CDC25C expression level was closely related to the grade and stage in human ovarian tumors and breast cancer.

When DNA damage occurs in cells, CDC25C acts as the main target of checkpoint kinase, is regulated by CHK1 and CHK2 by phosphorylation, and its activity is inhibited [17, 18]. CDC25 in the cytoplasm prevents cyclin B1/CDK1 complex activation and arrests the cell cycle in G2/M phase [19]. Activated CHK1 and CHK2 kinases phosphorylate CDC25C at Ser216 to promote the nuclear output of CDC25C. The gradually accumulated CDC25C in the cytoplasm eventually leads to G2/M arrest and is then degraded by ubiquitin-dependent degradation pathway [20]. Results of our study showed that CDC25 expression levels increased after MG132 treatment. In addition, CHK1 and CHK2 expressions were different in cells before and after CoCl_2_ treatment. PGCCs with the daughter cells in HEY and BT-549, highly expressed CHK2 and the CHK1 expression decreased. CHK1 and CHK2 in the nucleus can prevent CDC25C from dephosphorylating CDK1. Once CDK1 is activated, CHK1 is phosphorylated by CDK1 at Ser286 and Ser301 sites in turn. This phosphorylation induces nucleus to cytoplasm CHK1 translocation and eventually the accumulated cytoplasmic CHK1 is degraded by the proteasome degradation pathway [21, 22]. CHK2 is regulated by ATM after DNA double strand breaks and participates in the checkpoint regulation by phosphorylating CDC25C-Ser216. Compared with ATR-CHK1, ATM-CHK2 aims to provide a rapid protective response to DNA damage in cells [23, 24].

CDC25C phosphorylation can also be regulated by PLK1 and Aurora A. PLK1 expression level is different in different cell cycle stages and reaches the peak in the G2/M stage [25–27]. Activated PLK1 participates in cell cycle regulation by coordinating the nuclear translocation of M phase promoting factor (MPF) and its activator [28]. Aurora A regulates CDC25C–cyclin B1–CDK1 activation through PLK1 phosphorylation [29, 30]. Results of CDC25C knockdown showed that there might be a negative feedback loop between Aurora A, PLK1 expression, and CDC25C. Inhibition of CDC25C expression also affected Aurora A and PLK1 expressions. Current studies have confirmed that PLK1 carries CDC25C nuclear translocation signal and promotes CDC25C nuclear localization by phosphorylating CDC25C Ser198 site in G2/M phase [31, 32]. The phosphorylation of this site is also a necessary condition for regulating CDC25C nuclear translocation [33].

The expression of pCDC25CSer198 decreased in CoCl_2_-treated HEY PGCCs and increased in BT-549 PGCCs, which may be related to *p*53 genotype. Mutant *p53* not only cannot exert its anti-cancer function, but also affects wild-type *p53* normal function and promotes cancer progression [34]. CDC25C nuclear localization failure cannot activate cyclin B1–CDK1 complex and further initiate mitosis, which promotes PGCCs formation. The continuous activation of Aurora A–PLK1 can phosphorylate CDC25C-Ser198 and increase the phosphorylation level [35]. Hypoxia can increase mutant-type *p*53 expression, induce P53 protein accumulation and stabilization, reduce P53 degradation, and trigger *p*53-dependent apoptosis [36–38]. Beyfuss et al. have reported that hypoxia-induced P53 expression may be associated with increased Ser-37, Ser-46, and Ser-92 phosphorylation and decreased Lys373 acetylation [39]. In this study, CoCl_2_ could increase mutant-type *p53* expression and high P53 expression can inhibit CDK1, cyclin B1, and cyclin D1 expressions, which results in cell cycle G2/M arrest and PGCCs formation. In addition, co-immunoprecipitation and siRNA interference assays showed that P53 directly regulated pCDC25CSer216 and pCDC25CSer198 phosphorylation. P53 knockdown could reduce pCDC25CSer216 and pCDC25CSer198 expression. Kim et al. have reported that wide-type *p*53 could inhibit phosphorylation at CDC25C-Ser216 and prevent the polyploid generation caused by mitosis failure. However, mutant-type *p*53 decreased mitotic checkpoint function and increased CDC25C-Ser216 phosphorylation, which resulted in aneuploidy due to chromosome segregation failure [40]. In HEY, wild-type *p*53 decreased pCDC25CSer216 and pCDC25CSer198 expression levels. In BT-549, mutant *p*53 can promote PGCCs formation by mediating pCDC25CSer216 and pCDC25CSer198 phosphorylation level, increase tumor heterogeneity, and accelerate tumor progression.

## Conclusion

The abnormal expression and subcellular location of CDC25C associated with the formation of PGCCs. CHK1, CHK2–pCDC25CSer216–cyclin B1–CDK1 and Aurora A–PLK1–pCDC25CSer198–cyclin B1–CDK1 signaling pathways may participate in PGCCs formation and the various phosphorylation sites of CDC25C may depend on *p53* genotype.

### Supplementary information

**Additional file 1:** Supplementary figures and figure legends.

**Additional file 2.** Supplementary materials and methods.

**Additional file 3:** Supplementary tables.

## Data Availability

The authors declare that all data supporting the findings of this study are available within the article and its additional files or contact the corresponding author upon reasonable request.

## Abbreviations

CSC: Cancer stem cells
PGCCs: Polyploid giant cancer cells
CoCl_2_: Cobalt chloride
EMT: Epithelial-mesenchymal transition
DDRFBS: Fetal bovine serum
PBS: Phosphate-buffered saline
ICC: Immunocytochemistry
WB: Western blot
IHC: Immunohistochemistry
PMSF: Phenylmethanesulfonylfluoride fluoride
PI: Protease inhibitor cocktail
CDC25: Cell division cycle 25
CDKs: Cyclin-dependent protein kinases
CDI: Cyclin-dependent protein kinase inhibitor
CHK: Checkpoint kinase
ATR: Ataxia telangiectasia-mutated and Rad3-related homolog
ATM: Ataxia telangiectasia mutated
PLK1: Polo-like kinase 1
MPF: M-phase promoting factor
Ser: Serine
Thr: Threonine
Tyr: Tyrosine
