# Correction: Different *p53* genotypes regulating different phosphorylation sites and subcellular location of CDC25C associated with the formation of polyploid giant cancer cells

**DOI:** 10.1186/s13046-024-03155-z

**Published:** 2024-08-17

**Authors:** Kai Liu, Minying Zheng, Qi Zhao, Kexin Zhang, Zugui Li, Fangmei Fu, Hao Zhang, Jiaxing Du, Yuwei Li, Shiwu Zhang

**Affiliations:** 1 Department of Pathology, Tianjin Union Medical Center, Tianjin, 300121 P.R. China; 2 Graduate School, Tianjin Medical University, Tianjin, 300070 P.R. China; 3 Nankai University School of Medicine, Nankai University, Tianjin, 300071 P.R. China; 4 Graduate School, Tianjin University of Traditional Chinese Medicine, Tianjin, 300071 P.R. China; 5 Departments of Colorectal Surgery, Tianjin Union Medical Center, Tianjin, 300121 P.R. China

**Correction: BMC Plant Biol 39**,** 83 (2020)**

10.1186/s13046-020-01588-w

Following the publication of the original article [1], the authors identified errors in Fig. 3, specifically:

- Figure 3D - the β-actin of HEY and BT-549 control cells were mistakenly reusede.
- Figure 3E - the cytoplasm and nuclear protein expression of CDC25C in HEY and BT-549 control and PGCCs with daughter cells the same result was mistakenly placed.

Incorrect Fig. 3

**Fig. 3:**
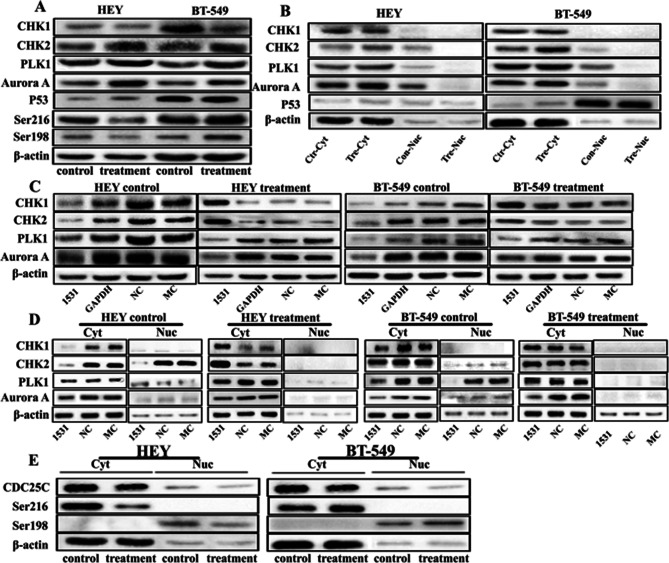
The expression of CHK1, CHK2, PLK1, Aurora A, P53, pCDC25CSer216, and pCDC25CSer198 in HEY and BT-549 control cells and PGCCs with budding daughter cells with and without CDC25C knockdown. **a** Western blot showed the total protein expression of CHK1, CHK2, PLK1, Aurora A, P53, pCDC25CSer216, and pCDC25CSer198 in HEY and BT-549 control and PGCCs with daughter cells. **b** The levels of total protein expression of CHK1, CHK2, PLK1, and Aurora A in HEY and BT-549 control and PGCCs with daughter cells, which were transfected with CDC25Ci, siRNA control, and negative control. **c** Cytoplasmic and nuclear protein expression of CHK1, CHK2, PLK1, Aurora A, P53 in HEY and BT-549 control and PGCCs with daughter cells. **d** Cytoplasmic and nuclear expression of CHK1, CHK2, PLK1, and Aurora A in HEY and BT-549 control and PGCCs with daughter cells, which were transfected with CDC25Ci, siRNA control, and negative control. **e** The cytoplasm and nuclear protein expression of pCDC25CSer216 and pCDC25CSer198 in HEY and BT-549 control and PGCCs with daughter cells. Treatment: Cells treated with CoCl2. 1531si: siRNA CDC25C-1531

Correct Fig. 3

**Fig. 3:**
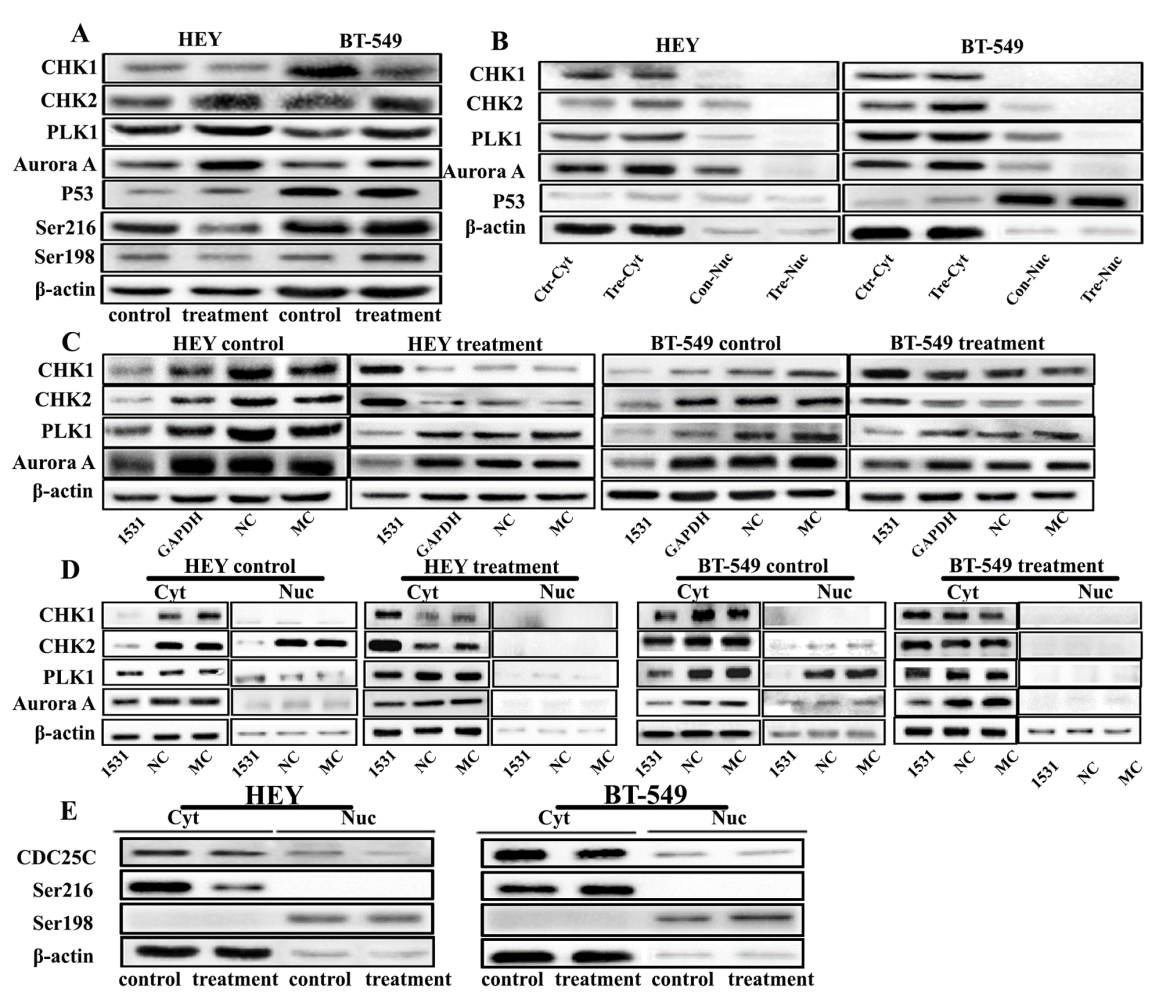
The expression of CHK1, CHK2, PLK1, Aurora A, P53, pCDC25CSer216, and pCDC25CSer198 in HEY and BT-549 control cells and PGCCs with budding daughter cells with and without CDC25C knockdown. **a** Western blot showed the total protein expression of CHK1, CHK2, PLK1, Aurora A, P53, pCDC25CSer216, and pCDC25CSer198 in HEY and BT-549 control and PGCCs with daughter cells. **b** The levels of total protein expression of CHK1, CHK2, PLK1, and Aurora A in HEY and BT-549 control and PGCCs with daughter cells, which were transfected with CDC25Ci, siRNA control, and negative control. **c** Cytoplasmic and nuclear protein expression of CHK1, CHK2, PLK1, Aurora A, P53 in HEY and BT-549 control and PGCCs with daughter cells. **d** Cytoplasmic and nuclear expression of CHK1, CHK2, PLK1, and Aurora A in HEY and BT-549 control and PGCCs with daughter cells, which were transfected with CDC25Ci, siRNA control, and negative control. **e** The cytoplasm and nuclear protein expression of pCDC25CSer216 and pCDC25CSer198 in HEY and BT-549 control and PGCCs with daughter cells. Treatment: Cells treated with CoCl2. 1531si: siRNA CDC25C-1531

The original article [1] has been corrected.
