# Supplementary material for: Different p53 genotypes regulating different phosphorylation sites and subcellular location of CDC25C associated with the formation of polyploid giant cancer cells
Source: J Exp Clin Cancer Res. 2020 May 11;39:83. doi: 10.1186/s13046-020-01588-w (PMC7212590; doi:10.1186/s13046-020-01588-w)
Supplement: Supplementary file 1 — Additional file 1: Supplementary figures and figure legends. [file 13046_2020_1588_MOESM1_ESM.pdf]

Figure S1

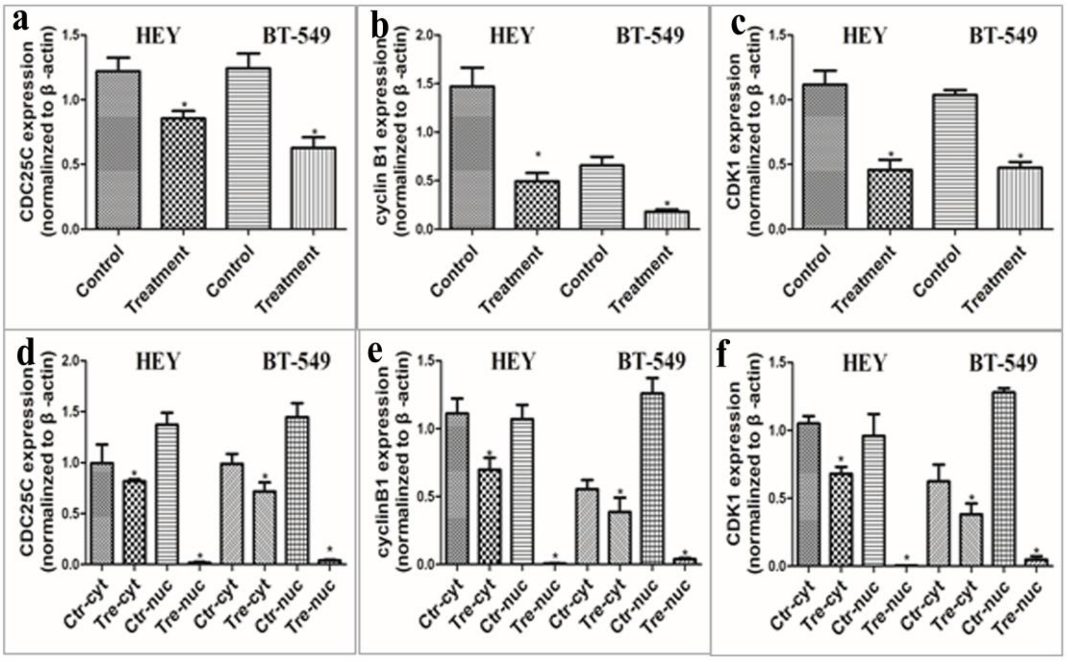

Supplementary figure 1. Quantitative results of total protein and cytoplasm and nuclear protein expression differences are shown as histograms. (a) The histogram of total CDC25C expression in HEY and BT-549. (b) The histogram of total cyclin B1 expression in HEY and BT-549. (c) The histogram of total CDK1 expression in HEY and BT-549. (d) The histogram of cytoplasm and nuclear CDC25C expression in HEY and BT-549. (e) The histogram of cytoplasm and nuclear cyclin B1 expression in HEY and BT-549. (f) The histogram of cytoplasm and nuclear CDK1 expression in HEY and BT-549.

Figure S2

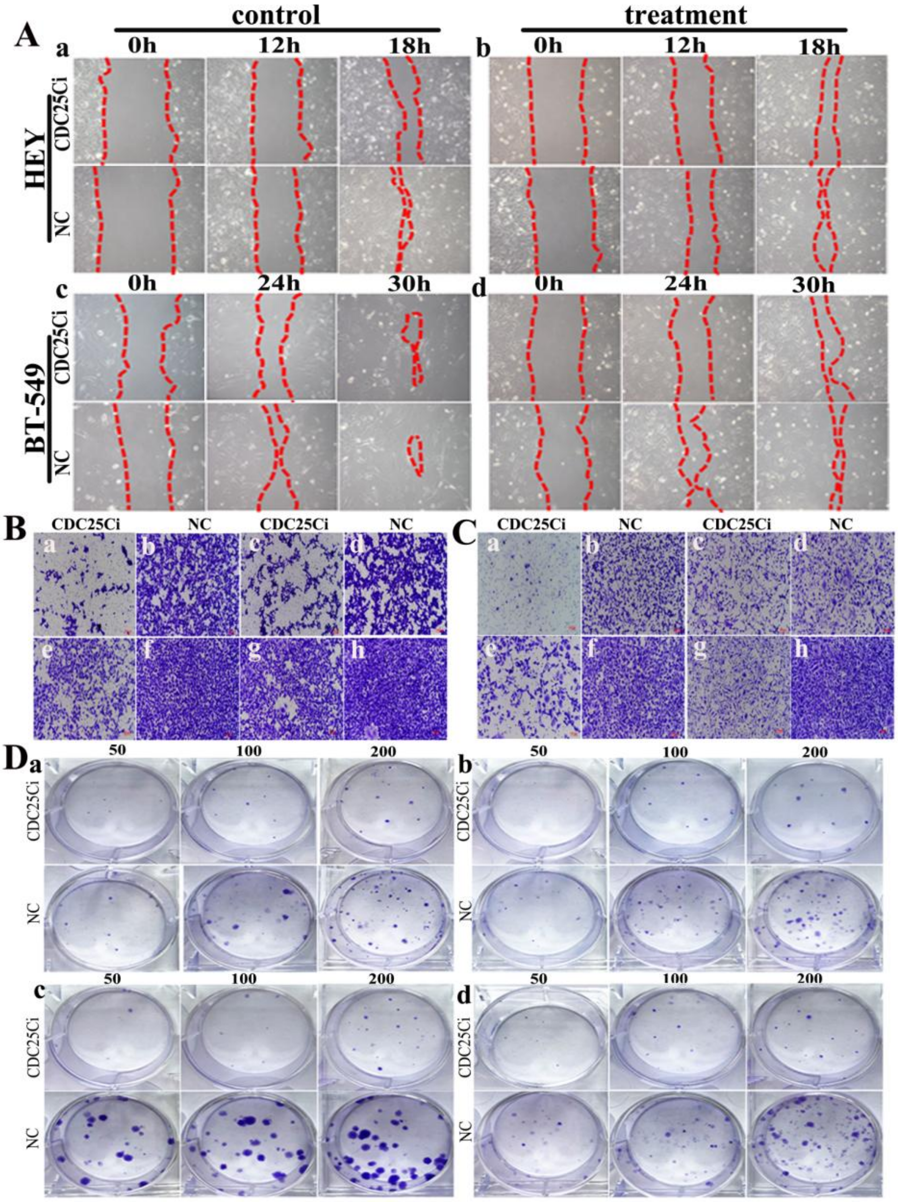

Supplementary figure 2. The migration, invasion, and proliferation ability of HEY and BT-549 cells after CDC25C knockdown. **A.** Wound-healing assay at different time period (100×). Wound-healing assays for (a) HEY control cells transfected with CDC25Ci and siRNA control, (b) HEY PGCCs with daughter cells transfected with CDC25Ci and siRNA control, (c) BT-549 control cells transfected with CDC25Ci and siRNA control, and (d) W BT-549 PGCCs with daughter cells transfected with CDC25Ci and siRNA control. **B.** Transwell migration assay showing the cells migration capacity (100×). HEY control cells transfected with (a) CDC25Ci and (b) siRNA control. HEY PGCCs with daughter cells (c) after CDC25C knockdown and (d) transfected with siRNA control. BT-549 control cells transfected with (e) CDC25Ci and (f) siRNA control. BT-549 PGCCs with daughter cells (g) after CDC25Ci and (h)

transfected with siRNA control. **C.** Comparison of invasion ability in cells with different transfection (100×). HEY control cells transfected with (a) CDC25Ci and (b) siRNA control. HEY PGCCs with daughter cells (c) after CDC25C knockdown and (d) transfected with siRNA control. BT-549 control cells transfected with (e) CDC25Ci and (f) siRNA control. BT-549 PGCCs with daughter cells (g) after CDC25Ci and (h) transfected with siRNA control. **D.** Plate clone formation experiment was used to compare the proliferation ability between the control and CDC25Ci. (a) Colony formation efficiency between 50, 100, and 200 (a) HEY CDC25Ci and NC; (b) HEY CDC25Ci and NC in PGCCs with their daughter cells; (c) BT-549 CDC25Ci and NC; and (d) BT-549 CDC25Ci and NC in PGCCs and their daughter cells.

Figure S3

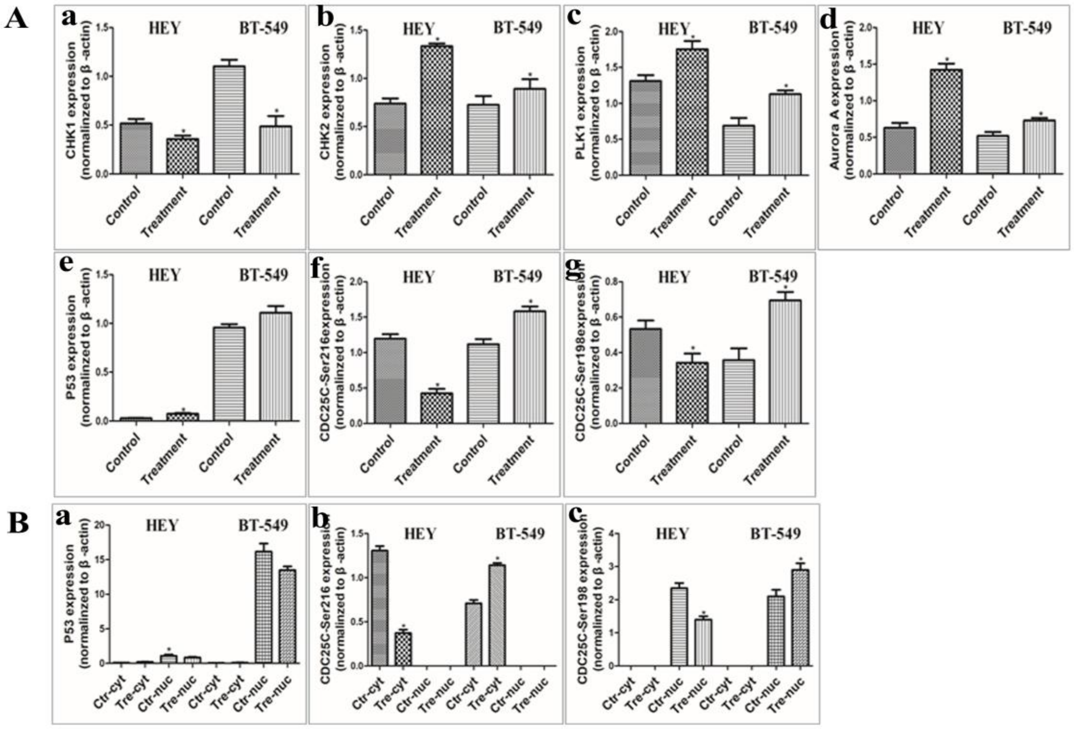

Supplementary figure 3. Quantitative results of total protein and cytoplasm and nuclear protein expression differences are shown as histograms. **A.** (a) The histogram of total CHK1 expression in HEY and BT-549. (b) The histogram of total CHK2 expression in HEY and BT-549. (c) The histogram of total PLK1 expression in HEY and BT-549. (d) The histogram of total Aurora A expression in HEY and BT-549. (e) The histogram of total P53 expression in HEY and BT-549. (f) The histogram of total CDC25C-Ser216 expression in HEY and BT-549. (g) The histogram of total CDC25C-Ser198 expression in HEY and BT-549. **B.** Quantitative results of differences are shown as histograms. (a) The histogram of and cytoplasm and nuclear protein P53 expression in HEY and BT-549. (b) The histogram of CDC25C-Ser216 expression in HEY and BT-549. (c) The histogram of CDC25C-Ser198 expression in HEY and BT-549.

Figure S4

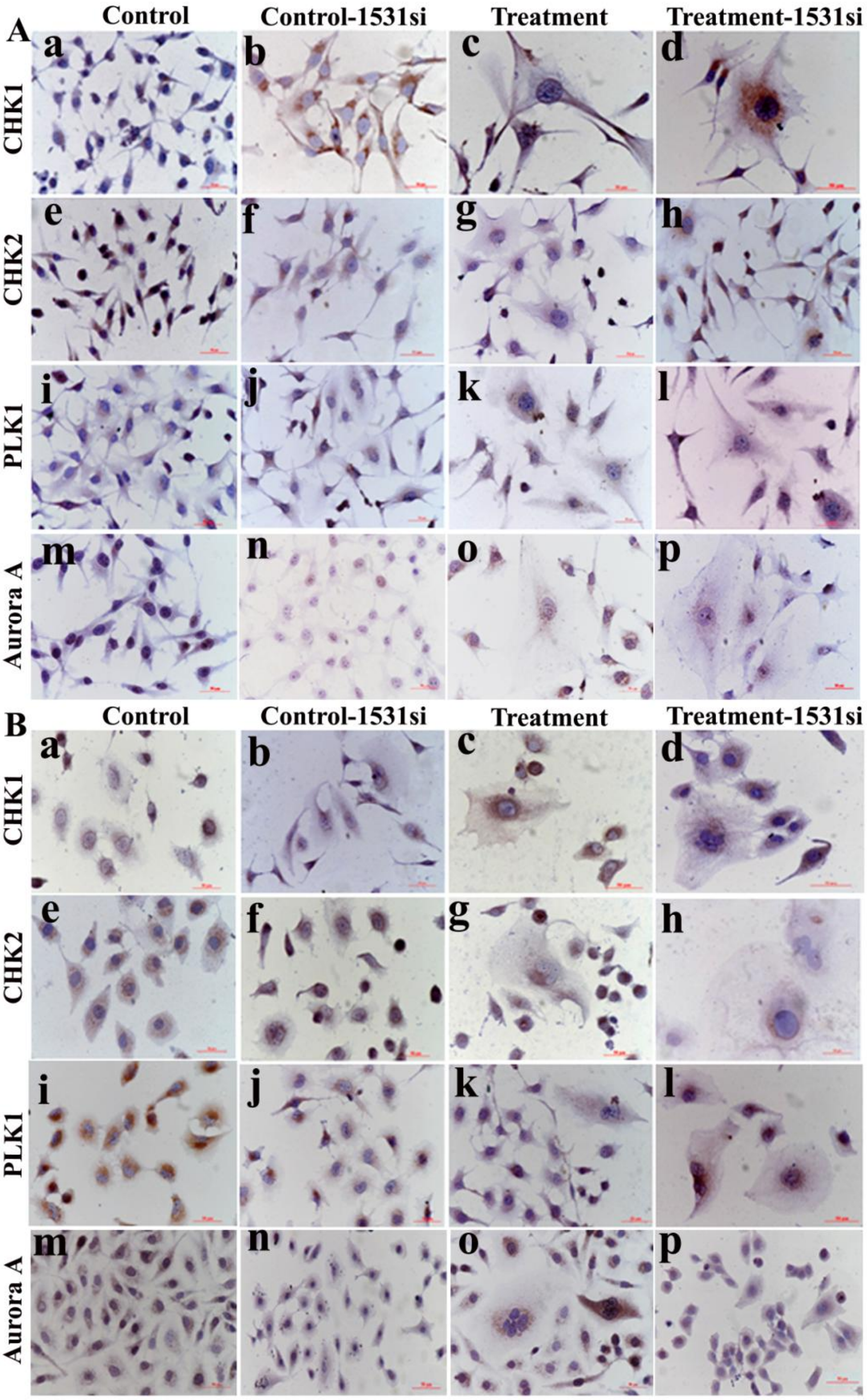

Supplementary figure 4. A. ICC staining of CHK1, CHK2, PLK1, Aurora A, P53, pCDC25C-Ser216 in HEY cells (200×). CHK1 in HEY (a) control cells, (b) control cells with CDC25Ci, (c) PGCCs with daughter cells, and (d) PGCCs with daughter

cells after CDC25C knockdown. CHK2 in HEY (e) control cells, (f) control cells with CDC25Ci, (g) PGCCs with daughter cells, and (h) PGCCs with daughter cells after CDC25C knockdown. PLK1 in HEY (i) control cells, (j) control cells with CDC25Ci, (k) PGCCs with daughter cells, and (l) PGCCs with daughter cells after CDC25C knockdown. Aurora A in HEY (m) control cells, (n) control cells with CDC25Ci, (o) PGCCs with daughter cells, and (p) PGCCs with daughter cells after CDC25C knockdown. **B.** ICC staining of CHK1, CHK2, PLK1, Aurora A, P53, pCDC25C-Ser216 in BT-549 cells (200×). CHK1 in BT-549 (a) control cells, (b) control cells with CDC25Ci, (c) PGCCs with daughter cells, and (d) PGCCs with daughter cells after CDC25C knockdown. CHK2 in BT-549 (e) control cells, (f) control cells with CDC25Ci, (g) PGCCs with daughter cells, and (h) PGCCs with daughter cells after CDC25C knockdown. PLK1 in BT-549 (i) control cells, (j) control cells with CDC25Ci, (k) PGCCs with daughter cells, and (l) PGCCs with daughter cells after CDC25C knockdown. Aurora A in BT-549 (m) control cells, (n) control cells with CDC25Ci, (o) PGCCs with daughter cells, and (p) PGCCs with daughter cells after CDC25C knockdown.

Figure S5

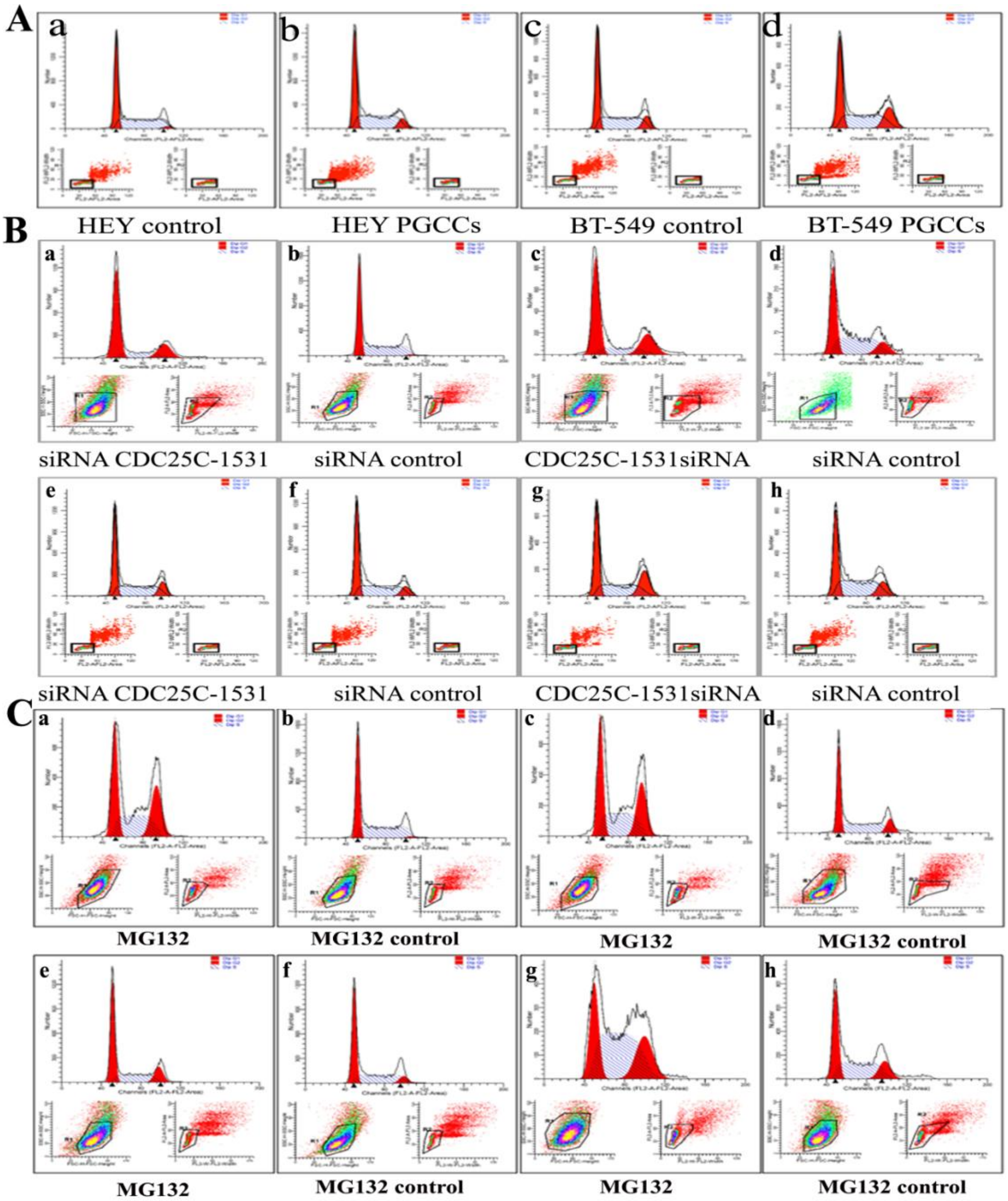

Supplementary figure 5. Flow cytometry analysis for cell cycle. **A.** There were more G2/M cells after $CoCl_2$ treatment. HEY (a) control and (b) PGCCs with daughter cells. BT-549 (c) control and (d) PGCCs with daughter cells. **B.** Cell cycle analysis after CDC25C knockdown. HEY control cells (a) after CDC25C knockdown and (b) transfected with siRNA control. HEY PGCCs with daughter cells (c) after CDC25C knockdown and (d) transfected with siRNA control. BT-549 control cells (e) after CDC25C knockdown and (f) transfected with siRNA control. BT-549 PGCCs with daughter cells (g) after CDC25C knockdown and (h) transfected with siRNA control.

C. Cell cycle analysis in G2/M phase after MG132 treatment. (a) HEY control cells. (b) HEY control cells after MG132 treatment. (c) HEY PGCCs with daughter cells. (d) HEY PGCCs with daughter cells after MG132 treatment. (e) BT-549 control cells. (f) BT-549 control cells after MG132 treatment. (g) BT-549 PGCCs with daughter cells. (h) BT-549 PGCCs with daughter cells after MG132 treatment.
