# Supplementary material for: Different p53 genotypes regulating different phosphorylation sites and subcellular location of CDC25C associated with the formation of polyploid giant cancer cells
Source: J Exp Clin Cancer Res. 2020 May 11;39:83. doi: 10.1186/s13046-020-01588-w (PMC7212590; doi:10.1186/s13046-020-01588-w)
Supplement: Supplementary file 2 — Additional file 2. Supplementary materials and methods. [file 13046_2020_1588_MOESM2_ESM.docx]

**Supplementary materials and methods**

**Transient siRNA transfection**

The CDC25C and P53 siRNA oligonucleotides were synthesized by Gene-pharma (Shanghai, China), including three siRNA interference sequences, one GAPDH positive control sequence, one negative control sequence, and one FAM negative control sequence. When the control cells and PGCCs with budding daughter cells were 60–70% confluent in six-well plates (2×10^5^ cells/well), Lipofectamine 2000 (Invitrogen, Carlsbad, CA, USA) and 1× Opti-MEM (Gibco, USA) were used to dilute the CDC25C/P53-negative control siRNA or CDC25C/P53 siRNA following the manufacturer’s protocol, and the mixture was added to the cells. The cells were harvested for 24–48 h after transfection to examine the effect of targeted protein knockdown with western blots. Detailed information about the siRNA oligonucleotide sequences is provided in the Supplementary table 1 and 2.

**Total protein extraction and separation of cytoplasmic nuclear proteins**

Total proteins were lysed on ice with a radioimmunoprecipitation assay (RIPA) lysis buffer for 30 min and centrifuged at 14,000 rpm for 30 min at 4 ℃. The cytoplasmic and nuclear proteins were isolated according to the manufacturer instructions (Beyotime Biotechnology, Shanghai, China). Briefly, the cells were collected and 200 µl reagent A (containing PMSF) was added to 20 µl cell pellets. After thorough mixing, 10 µl reagent B was added and the cytoplasmic proteins were immediately pipetted into a pre-cooled tube for use. The supernatant was thoroughly discarded to avoid cytoplasmic protein contamination and 50 µl nuclear protein extraction reagent (containing PMSF) was added to the precipitate. After vortex and centrifuge, the nuclear proteins were immediately collected in a new pre-cooled tube for assay.

**Western blot analysis**

The protein concentrations from HEY and BT-549 cells before and after CoCl_2_ treatment and CDC25C knockdown (CDC25Ci) were determined. A sodium dodecyl sulfate (SDS) polyacrylamide gel (10%) was used to separate the proteins, which were transferred to a polyvinylidene fluoride (PVDF) membrane (GE Healthcare, USA). The PVDF membrane was blocked with 5% milk in 1× Tris-buffered saline with 1% Tween-20 (TBST; Sigma-Aldrich) for 2 h at room temperature and then incubated with primary antibodies (Detail information listed in supplementary table 3) at 4 ℃ overnight. The membranes were further incubated with the secondary antibodies at 20–25 ℃ for 2 h. The final protein expression was detected by the Chemidoc imaging system (BioRad, USA). The Image-J software was used to detect the optical density of each band. The protein-loading control adopted β-actin and all western blot results were repeated more than thrice.

**H&E staining**

Control cells, PGCCs with daughter cells, and their corresponding CDC25Ci were cultured on slides for H&E staining. After fixation with 75% ethanol, these cells were stained with hematoxylin (Baso, Zhuhai, Guangzhou, China) for 1 min and eosin for 2 min.

**Immunohistochemical (IHC) and immunocytochemical (ICC) staining**

For IHC staining, tumor tissues were fixed with formalin, embedded in paraffin, and then sectioned. The sections were deparaffinized in xylene and dehydrated with a series of gradient ethanol solutions. Heated citrate buffer solution (pH 6.0) was used for antigen retrieval in an autoclave at 100 ℃ for 15 min. Then, 3% hydrogen peroxide was used for 15 min to block endogenous peroxidase activity, after rinsing the slides with PBS for 5 min thrice, and 10% goat serum was used to block nonspecific binding sites for 20 min. The slides were incubated with the primary antibodies (supplementary table 3) overnight at 4 ℃. After incubation, slides were washed with PBS and treated with biotinylated IgG and horseradish peroxidase-labeled streptomycin for 20 and 15 min, respectively. Finally, the slides were stained with 3,3’-diaminobenzidine (DAB) for 1–5 min, counterstained with hematoxylin for 30 s, and washed with running water. Images were captured under a microscope (Nikon Eclipse 80i, Japan). For ICC staining, slides with HEY and BT-549 PGCCs with budding daughter cells and control cells before and after treatment with CDC25Ci were fixed with ice-cold methyl alcohol for 30 min and incubated with primary antibodies at 4 ℃ for 16 h, secondary antibodies for 20 min, and horseradish peroxidase-labeled streptomycin (Zhongshan Inc, China) for 15 min at 37 ℃. The cells were then counterstained with hematoxylin.

**Scoring and quantification of IHC**

Yellow staining in the cytoplasm and/or nuclear was considered as positive and both the staining intensity and percentage of positive cells were evaluated. The staining intensity was scored as follows: 0, negative (no staining); 1, weak positive (light yellow staining); 2, moderate positive (brownish yellow staining); and 3, strong positive (brown staining). The percentage of positive cells was scored as follows: 0 (negative): less than 5% positive cells; 1 (weak): 6–30% positive cells; 2 (moderate): 31–50% positive cells; and 3 (strong): 51–100% positive cells. The sum of the staining intensity and positive cell scores was used to determine the staining index for each section.

**Cell migration and invasion assay**

Wound-scratch and transwell migration assays were used to detect the migration ability of the control cells and PGCCs with their daughter cells before and after transfection. For wound-scratch, cells were seeded into 12-well plates (three replicate wells per group) and cultured until 100% monolayer confluence was reached. Then, sterile pipette tips were used to uniformly scratch the monolayer cells vertically to form the wound tracks. After rinsing with PBS, the cells were cultured in serum-free medium. The migration ability was evaluated by photographing the wound area at 0, 12, and 18 h for HEY and 0, 24, and 30 h for BT-549 at the same scratch position. The Image-J software was used to outline the migration area and calculate the wound-healing index according to the following formula: [(the wound area at 0 h)−(the wound area at indicated time)]/(the wound area at 0 h). A high score indicated a strong migration ability.

For transwell migration assays, the cell culture inserts (8 μm; BD-Falcon, Franklin Lakes, NJ, USA) was placed into a 24-well plate. Cells (5×10^4^ cells per insert) in 200 μL medium without FBS were cultured in the upper chambers and the medium containing 20% FBS was added to the lower chamber. HEY and BT-549 cells were then incubated for 12 and 24 h, respectively, in a 37 ℃ incubator. Non-migrated cells were wiped off with wet cotton tips and cells that successfully migrated to the lower chamber membrane were fixed in cold methanol for 30 min. Finally, cells were stained with 0.1% crystal violet for 30 minutes. Images were captured at 100× magnification and the cells were counted from at least five different fields of view. Three independent experiments were performed. Cell invasion ability was determined by transwell invasion assays and the inserts were precoated with BD Matrigel Basement Membrane Matrix (Corning) in a 24-well plate. Cells (5×10^5^ cells per insert) in 200 μL medium without FBS were cultured in the upper chamber and the medium containing 20% FBS was added to the lower chamber. Then HEY and BT-539 cells were incubated for 12 and 24 h, respectively, in a 37 ℃ incubator. The invading cells were stained with crystal violet and photographed.

**Plate clone formation experiment**

PGCCs with budding daughter cells and control cells before and after CDC25i from HEY and BT-549 cells were counted with a cell counter. Cell suspensions (2 mL/well) with 50 cells, 100 cells, and 200 cells were cultured in the six-well plate and the plates were incubated for 1–2 weeks in a 37 ℃ cell incubator. The incubation was stopped when a white cell clone was visible. The cell clones were washed with PBS and fixed with cold methanol for 30 min. After staining with 0.1% crystal violet for 30 minutes, the number of cell clone group per well was counted under the microscope (the number of cells in a single clone should be more than 50) and the efficiency of colony formation was calculated by the following formula: formation efficiency = number of clones/number of cells inoculated. A high score indicated strong proliferation ability.

**Flow cytometry analysis of cell cycle**

Cells (1×10^6^) with different treatment were collected, trypsinized, then washed with cold PBS, and centrifuged at 1000 rpm for 10 min. Cold 75% ethanol was added to the cells dropwise with gentle vortex and incubated in dark at 4 ℃. The cells were washed with cold PBS twice to remove the ethanol and stained with 0.5 mL PI/RNase staining solution (BD Pharmingen PI/RNase staining buffer) and incubated at room temperature in dark for 15 min. Cell cycle was analyzed by flow cytometry on the FACSCalibur (BD Biosciences, USA). CellQuest (BD Biosciences, USA) and Modfit LT software were used to analyze the data.

**Vitro kinase activity assay**

The kinase activity assay was performed using the EnzyChrom Kinase Assay Kit (BioAssay Systems, USA) to test the differences in protein kinase activity before and after CoCl_2_ treatment in vitro. In this study, a protein concentration gradient was constructed and serine was used as the substrate for detection. The mixture containing the protein substrate, kinase, and ATP was incubated at room temperature for 30 min and a mixture containing only ATP and substrate was used as the blank control group. After incubation, the working solution was added to each well and the ADP was detected fluorescently after 10 min. Three duplicate wells were set for each group. The fluorescence intensity was detected using a multifunction microplate reader (BioTek) at 530 nm excitation and 590 nm emission. The kinase activity was calculated using the following equation:

$$Kinase Activity (U/L)= \frac{\Delta\mathrm{Fsample}}{\mathrm{Slope}\cdot t}\times\frac{100 \mu l}{Vol(\mu l)}$$

**Co-immunoprecipitation (Co-IP)**

Co-IP was performed to determine the direct or indirect interaction with pCDC25C-Ser216 and pCDC25C-Ser198 in HEY and BT-549 cells after CoCl_2_ treatment. The cells were washed with cold PBS and lysed with 600 μL IP lysis buffer (Thermo Fisher Scientific, Inc.) with 1× Halt Protease & Phosphatase Inhibitor Cocktail (Thermo Fisher Scientific, Inc.) for 30 mins on ice, followed by centrifugation at 14,000× *g* for 10 mins. Rabbit anti-pCDC25C-Ser216 and anti-pCDC25C-Ser198 monoclonal antibody (4 μg; 1:50 dilution) were incubated with samples at 4 °C overnight and 4 μg normal rabbit IgG (Beyotime, Shanghai, China) was used as a negative control. A total of 30 μL pre-washed protein A/G agarose beads (Thermo Fisher Scientific, Inc.) were added to the mixture and rolled for 2 h at 4 °C. After washing and centrifuging, the immunoprecipitants were examined by silver staining and western blot using anti-pCDC25C-Ser216 and anti-pCDC25C-Ser198.
