# Supplementary material for: Different p53 genotypes regulating different phosphorylation sites and subcellular location of CDC25C associated with the formation of polyploid giant cancer cells
Source: J Exp Clin Cancer Res. 2020 May 11;39:83. doi: 10.1186/s13046-020-01588-w (PMC7212590; doi:10.1186/s13046-020-01588-w)
Supplement: Supplementary file 3 — Additional file 3: Supplementary tables. [file 13046_2020_1588_MOESM3_ESM.doc]

Supplementary table 1. CDC25C-siRNA interfering sequences.

| Names | Sense (5ʹ-3ʹ) | Antisense (5ʹ-3ʹ) |
| --- | --- | --- |
| CDC25Ci-1103 | GUCUCUCUGUGUGACAUUATT | UAAUGUCACACAGAGAGACTT |
| CDC25Ci-1343 | CAGGGAGCCUUAAACUUAUTT | AUAAGUUUAAGGCUCCCUGTT |
| CDC25Ci-1531 | GUACUACCCAGAGCUAUAUTT | AUAUAGCUCUGGGUAGUACTT |
| CDC25Ci-GAPDH | UGACCUCAACUACAUGGUUTT | AACCAUGUAGUUGAGGUCATT |
| CDC25Ci-NC | UUCUCCGAACGUGUCACGUTT | ACGUGACACGUUCGGAGAATT |

Supplementary table 2. p53-siRNA interfering sequences.

| Names | Sense (5ʹ-3ʹ) | Antisense (5ʹ-3ʹ) |
| --- | --- | --- |
| TP53i-339 | CCGGACGAUAUUGAACAAUTT | AUUGUUCAAUAUCGUCCGGTT |
| TP53i-886 | GUACCACCAUCCACUACAATT | UUGUAGUGGAUGGUGGUACTT |
| TP53i-985 | GUAAUCUACUGGGACGGAATT | UUCCGUCCCAGUAGAUUACTT |
| TP53i-GAPDH | UGACCUCAACUACAUGGUUTT | AACCAUGUAGUUGAGGUCATT |
| TP53i-NC | UUCUCCGAACGUGUCACGUTT | ACGUGACACGUUCGGAGAATT |

Supplementary table 3. Antibodies used in this study (western blot and ICC).

| Reagent | Species Specificity | Company | WB | ICC | | IHC |
| --- | --- | --- | --- | --- | --- | --- |
| CDC25C | Rabbit monoclonal | Abcam | 1:3000 | 1:500 | 1:1000 | |
| Cyclin B1 | Rabbit polyclonal | Proteintech | 1:3000 | 1:500 | 1:1000 | |
| CDK1 | Rabbit monoclonal | Abcam | 1:3000 | 1:500 | 1:1000 | |
| CHK1 | Rabbit polyclonal | Proteintech | 1:1000 | 1:100 | 1:400 | |
| CHK2 | Rabbit polyclonal | Abcam | 1:1000 | 1:100 | 1:300 | |
| PLK1 | Mouse monoclonal | Origene | 1:1000 | 1:100 | 1:400 | |
| Aurora A | Mouse polyclonal | Proteintech | 1:1000 | 1:100 | 1:400 | |
| P53 | Rabbit polyclonal | Proteintech | 1:3000 | 1:2000 | 1:5000 | |
| pCDC25C-Ser216 | Rabbit monoclonal | CST | 1:500 | 1:100 | 1:100 | |
| pCDC25C-Ser198 | Rabbit monoclonal | CST | 1:500 |  |  | |
| β-actin | Mouse monoclonal | Proteintech | 1:1000 |
| GAPDH | Mouse monoclonal | Proteintech | 1:3000 |

Supplementary table 4.The groups and clinical features of ovarian and breast cancers.

| Tumor | Group | Clinical feature |
| --- | --- | --- |
| Breast cancer | GroupⅠ | Invasive breast cancer with lymph node metastasis |
|  | GroupⅡ | Invasive breast cancer without lymph node metastasis |
| Ovarian tumor | Group Ⅰ | primary serous ovarian cancer with lymph node metastasis |
|  | Group Ⅱ | lymph node metastatic foci of Group Ⅰ |
|  | Group Ⅲ | primary serous ovarian cancer without lymph node metastasis |
|  | Group Ⅳ | borderline serous cystadenoma |

Supplementary table 5. The percentage of PGCCs in HEY and BT-549 cells before and after CoCl_2_ treatment and CDC25C-siRNA transfection (H&E)

|  |  | Rate of PGCCs |  | t | *P* value |
| --- | --- | --- | --- | --- | --- |
| HEY | Control | 0.0041±0.0026 | | −12.146 | 0.001 |
|  | Ctr-CDC25i | 0.0289±0.0074 | |  |  |
|  | PGCCs | 0.1215±0.0115 | | −9.9373 | 0.000 |
|  | PGCCs-CDC25i | 0.2773±0.0331 | |  |  |
| BT-549 | Control | 0.0090±0.0042 | | −9.8896 | 0.000 |
|  | Ctr- CDC25i | 0.0275±0.0069 | |  |  |
|  | PGCCs | 0.1379±0.0247 | | −4.8222 | 0.000 |
|  | PGCCs-CDC25i | 0.2366±0.0385 | |  |  |

CDC25Ci is used to represent siRNA CDC25C-1531.

Supplementary table 6. The percentage of wound-healing index in HEY cells before and after CoCl_2_ treatment and CDC25C-siRNA transfection.

|  |  | Wound-healing index (%) | t | *P* value |
| --- | --- | --- | --- | --- |
| HEY-Con | Ctr-CDC25Ci-12h | 0.3613±0.0269 | −6.0657 | 0.006 |
|  | Ctr-NCi-12h | 0.4974±0.0167 |  |  |
|  | Ctr-CDC25Ci-18h | 0.8155±0.0568 | −3.9711 | 0.022 |
|  | Ctr-NCi-18h | 1.0004±0.0355 |  |  |
| HEY-Tre | Tre-CDC25Ci-12h | 0.4871±0.0258 | −5.4212 | 0.015 |
|  | Tre -NCi-12h | 0.7264±0.3068 |  |  |
|  | Tre -CDC25Ci-18h | 0.9103±0.0501 | −3.6867 | 0.0407 |
|  | Tre -NCi-18h | 1.108±0.5682 |  |  |

Supplementary table 7. The percentage of wound-healing index in BT-549 cells before and after CoCl_2_ treatment and CDC25C-siRNA transfection.

|  |  | Wound-healing index (%) | t | *P* value |
| --- | --- | --- | --- | --- |
| BT-549-Con | Ctr-CDC25Ci-24h | 0.2477±0.0279 | −9.8847 | 0.001 |
|  | Ctr-NCi-24h | 0.5153±0.1927 |  |  |
|  | Ctr-CDC25Ci-30h | 0.6438±0.0193 | −5.9260 | 0.005 |
|  | Ctr-NCi-30h | 0.7843±0.0474 |  |  |
| BT-549-Tre | Tre-CDC25Ci-24h | 0.4060±0.0241 | −4.1096 | 0.017 |
|  | Tre -NCi-24h | 0.5196±0.0773 |  |  |
|  | Tre -CDC25Ci-30h | 0.7033±0.3003 | −3.7437 | 0.041 |
|  | Tre -NCi-30h | 0.9040±0.0684 |  |  |

Supplementary table 8. The average migration field in HEY and BT-549 cells before and after CoCl_2_ treatment and CDC25C-siRNA transfection.

|  |  | Average cell migration | t | *P* value |
| --- | --- | --- | --- | --- |
| HEY | Ctr-CDC25Ci | 87.6667±13.0725 | −9.8847 | 0.001 |
|  | Ctr-NC | 257.0000±26.1916 |  |  |
|  | Tre-CDC25Ci | 159.6667±20.8540 | −5.9260 | 0.005 |
|  | Tre-NC | 394.3333±20.8859 |  |  |
| BT-549 | Ctr-CDC25Ci | 279.0000±23.1567 | −10.89 | 0.001 |
|  | Ctr-NC | 625.3333±38.5602 |  |  |
|  | Tre-CDC25Ci | 386.3333±13.7194 | −26.6 | 0.000 |
|  | Tre-NC | 781.6667±15.9234 |  |  |

Supplementary table 9. The average invasion field in HEY and BT-549 cells before and after CoCl_2_ treatment and CDC25C-siRNA transfection.

|  |  | Average cell invasion | t | *P* value |
| --- | --- | --- | --- | --- |
| HEY | Ctr-CDC25Ci | 31.6667±6.1283 | −16.442 | 0.002 |
|  | Ctr-NC | 266.0000±19.2007 |  |  |
|  | Tre-CDC25Ci | 229.3333±15.1511 | −6.3607 | 0.010 |
|  | Tre-NC | 401.3333±35.1125 |  |  |
| BT-549 | Ctr-CDC25Ci | 211.0000±18.3848 | −13.187 | 0.001 |
|  | Ctr-NCi | 556.6667±32.1904 |  |  |
|  | Tre-CDC25Ci | 307.3333±20.9815 | −19.759 | 0.000 |
|  | Tre-NC | 708.6667±19.6186 |  |  |

Supplementary table 10. The percentage of colony information efficiency in HEY and BT-549 cells before and after CoCl_2_ treatment and CDC25C-siRNA transfection.

|  |  | Colony information efficiency (%) | t | *P* value |
| --- | --- | --- | --- | --- |
| HEY | Ctr-CDC25Ci | 14.6667±5.3125 | −7.9383 | 0.001 |
|  | Ctr-NC | 56.0000±5.0990 |  |  |
|  | Tre-CDC25Ci | 26.0000±5.0910 | −8.5190 | 0.004 |
|  | Tre-NC | 99.6667±11.1156 |  |  |
| BT-549 | Ctr-CDC25Ci | 19.3333±2.6247 | −7.7169 | 0.005 |
|  | Ctr-NC | 51.6667±5.3125 |  |  |
|  | Tre-CDC25Ci | 33.0000±4.5460 | −14.1 | 0.000 |
|  | Tre-NC | 87.0000±2.9439 |  |  |

Supplementary table 11. Differences of CDC25C expression in human breast and ovarian cancer tissues

| Tumor | Group | n | Staining index for CDC25C | Value of statistic | *P* |
| --- | --- | --- | --- | --- | --- |
| Breast cancer | GroupI | 62 | 6.72±2.58 | Z= −5.097 | 0.001 |
|  | GroupII | 167 | 3.77±2.63 |  |  |
| Ovarian cancer | Group Ⅰ | 20 | 4.55±2.66 | χ^2^= 35.97 | 0.000^a^ |
|  | Group Ⅱ* | 20 | 7.50±2.52 |  |  |
|  | Group Ⅲ** | 31 | 3.54±2.36 |  |  |
|  | Group Ⅳ*** | 10 | 0.80±1.32 |  |  |

*: Indicates the comparison between two groups

*: Comparison of Group Ⅱ vs. Group Ⅰ

**: Comparison of Group Ⅲ vs. Group Ⅰ

***: Comparison of Group Ⅳ vs. Group Ⅲ

Supplementary table 12. Differences of CDK1 expression in human breast and ovarian cancer tissues

| Tumor | Group | N | Staining index for CDK1 | Value of statistic | *P* |
| --- | --- | --- | --- | --- | --- |
| Breast cancer | GroupⅠ | 62 | 6.68±2.41 | Z= −5.98 | 0.000 |
|  | GroupⅡ | 167 | 2.94±2.21 |  |  |
| Ovarian cancer | Group Ⅰ | 20 | 4.95±2.14 | χ^2^= 44.94 | 0.000^a^ |
|  | Group Ⅱ* | 20 | 7.45±1.85 |  |  |
|  | Group Ⅲ** | 31 | 3.13±2.26 |  |  |
|  | Group Ⅳ*** | 10 | 0.20±0.63 |  |  |

*: Indicates the comparison between two groups

*: Comparison of Group Ⅱ vs. Group Ⅰ

**: Comparison of Group Ⅲ vs. Group Ⅰ

***: Comparison of Group Ⅳ vs. Group Ⅲ

Supplementary table 13. Differences of CHK1 expression in human breast and ovarian cancer tissues

| Tumor | Group | N | Staining index for CHK1 | Value of statistic | *P* |
| --- | --- | --- | --- | --- | --- |
| Breast cancer | GroupⅠ | 62 | 7.42±2.23 | Z= −5.67 | 0.001 |
|  | GroupⅡ | 167 | 4.22±2.52 |  |  |
| Ovarian cancer | Group Ⅰ | 20 | 5.40±2.28 | χ^2^= 32.94 | 0.000^a^ |
|  | Group Ⅱ* | 20 | 7.30±2.31 |  |  |
|  | Group Ⅲ** | 31 | 4.17±2.63 |  |  |
|  | Group Ⅳ*** | 10 | 0.90±2.02 |  |  |

*: Indicates the comparison between two groups

*: Comparison of Group Ⅱ vs. Group Ⅰ

**: Comparison of Group Ⅲ vs. Group Ⅰ

***: Comparison of Group Ⅳ vs. Group Ⅲ

Supplementary table 14. Differences of CHK2 expression in human breast and ovarian cancer tissues

| Tumor | Group | N | Staining index for CHK2 | Value of statistic | *P* |
| --- | --- | --- | --- | --- | --- |
| Breast cancer | GroupⅠ | 62 | 6.76±2.57 | Z= −6.56 | 0.000 |
|  | GroupⅡ | 167 | 2.70±2.36 |  |  |
| Ovarian cancer | Group Ⅰ | 20 | 4.90±2.73 | χ^2^= 41.35 | 0.000^a^ |
|  | Group Ⅱ* | 20 | 8.15±2.23 |  |  |
|  | Group Ⅲ** | 31 | 4.35±2.16 |  |  |
|  | Group Ⅳ*** | 10 | 0.40±1.26 |  |  |

*: Indicates the comparison between two groups

*: Comparison of Group Ⅱ vs. Group Ⅰ

**: Comparison of Group Ⅲ vs. Group Ⅰ

***: Comparison of Group Ⅳ vs. Group Ⅲ

Supplementary table 15. Differences of PLK1 expression in human breast and ovarian cancer tissues

| Tumor | Group | N | Staining index for PLK1 | Value of statistic | *P* |
| --- | --- | --- | --- | --- | --- |
| Breast cancer | Group Ⅰ | 62 | 7.50±2.53 | Z= −3.88 | 0.000 |
|  | Group Ⅱ | 167 | 4.23±2.86 |  |  |
| Ovarian cancer | Group Ⅰ | 20 | 4.55±2.83 | χ^2^= 39.47 | 0.000^a^ |
|  | Group Ⅱ* | 20 | 7.75±2.40 |  |  |
|  | Group Ⅲ | 31 | 3.55±2.46 |  |  |
|  | Group Ⅳ*** | 10 | 0.40±1.26 |  |  |

*: Indicates the comparison between two groups

*: Comparison of Group Ⅱ vs. Group Ⅰ

***: Comparison of Group Ⅳ vs. Group Ⅲ

Supplementary table 16. Differences of Aurora A expression in human breast and ovarian cancer tissues

| Tumor | Group | N | Staining index for Aurora A | Value of statistic | *P* |
| --- | --- | --- | --- | --- | --- |
| Breast cancer | GroupⅠ | 62 | 5.94±2.69 | Z= −4.86 | 0.000 |
|  | GroupⅡ | 167 | 3.58±2.31 |  |  |
| Ovarian cancer | Group Ⅰ | 20 | 5.15±2.58 | χ^2^= 33.28 | 0.000^a^ |
|  | Group Ⅱ* | 20 | 7.60±2.30 |  |  |
|  | Group Ⅲ | 31 | 4.25±2.40 |  |  |
|  | Group Ⅳ*** | 10 | 1.2±0.943 |  |  |

*: Indicates the comparison between two groups

*: Comparison of Group Ⅱ vs. Group Ⅰ

***: Comparison of Group Ⅳ vs. Group Ⅲ
